# Oncolytic potency and reduced virus tumor-specificity in oncolytic virotherapy. A mathematical modelling approach

**DOI:** 10.1371/journal.pone.0184347

**Published:** 2017-09-21

**Authors:** Khaphetsi Joseph Mahasa, Amina Eladdadi, Lisette de Pillis, Rachid Ouifki

**Affiliations:** 1 DST/NRF Centre of Excellence in Epidemiological Modelling and Analysis (SACEMA), University of Stellenbosch, Stellenbosch, South Africa; 2 The College of Saint Rose, Albany, NY, United States of America; 3 Harvey Mudd College, Claremont, CA, United States of America; 4 Department of Mathematics and Applied Mathematics, University of Pretoria, Pretoria, South Africa; University of California Irvine, UNITED STATES

## Abstract

In the present paper, we address by means of mathematical modeling the following main question: How can oncolytic virus infection of some normal cells in the vicinity of tumor cells enhance oncolytic virotherapy? We formulate a mathematical model describing the interactions between the oncolytic virus, the tumor cells, the normal cells, and the antitumoral and antiviral immune responses. The model consists of a system of delay differential equations with one (discrete) delay. We derive the model’s basic reproductive number within tumor and normal cell populations and use their ratio as a metric for virus tumor-specificity. Numerical simulations are performed for different values of the basic reproduction numbers and their ratios to investigate potential trade-offs between tumor reduction and normal cells losses. A fundamental feature unravelled by the model simulations is its great sensitivity to parameters that account for most variation in the early or late stages of oncolytic virotherapy. From a clinical point of view, our findings indicate that designing an oncolytic virus that is not 100% tumor-specific can increase virus particles, which in turn, can further infect tumor cells. Moreover, our findings indicate that when infected tissues can be regenerated, oncolytic viral infection of normal cells could improve cancer treatment.

## Introduction

Oncolytic virotherapy is an emerging anti-cancer treatment modality that uses Oncolytic Viruses (OVs). One of the most attractive features of the OVs is that they are either naturally occurring or genetically engineered to selectively infect, replicate in and damage tumor cells while leaving normal cells intact [1, 2]. This therapeutic approach faces a major challenge consisting of the immune system’s response to the virus, which hinders oncolytic virotherapy. To date, complex dynamics of oncolytic viral tumor infection and the consequences of OV-induced immune response are poorly understood [3–5]. The immune system has often being perceived as a major impediment to successful oncolytic virus therapy by facilitating viral clearance [6, 7]. Additionally, clinical evidence [8–10] indicates that some oncolytic viruses have the ability to infect and replicate within normal cells as well, especially in the brain, where neurons are unable to replicate, and the oncolytic-induced neuronal damage could lead to undesired outcomes [11]. Evidence from both pre-clinical and clinical experiments indicates that some oncolytic viruses (OVs) can infect and replicate in normal cells surrounding the tumor [7, 12].

While this could be seen as another challenge to virotherapy, it could also be used to increase viral potency as long as the replication within normal cells is well understood and controlled. Much remains unknown about how to use normal cells to augment the oncolytic virus population [13, 14]. It is important to note that when systemically administering oncolytic virus that is not 100% tumor specific (i.e., viruses that can infect and replicate within normal cells), infection of some normal cells can occur [9, 10]. When administering oncolytic viruses intravenously, the amount of virions that effectively reach the tumor site is often reduced [15]. Note that viruses are small passive particles that reach their target cells via either radial cell-to-cell spread or diffusion across concentration gradients in soluble matters, such as blood, and propagate infection. Thus, infecting some normal cells, by oncolytic virus, surrounding the tumor may aid to increase virus population. The higher the number of infectious virions at the tumor territory, the higher the probability of infecting and destroying every single tumor cell [15, 16]. It is important to investigate how infection of the host normal cells by the OVs can enhance the oncolytic virotherapy. To normal cells, such as liver, that can be quickly self-regenerated after a trauma or disease, infection of normal cells could be tolerable if such infection is not endemic (i.e., the infection does not persist forever) and could potentially aid to control tumor growth [17].

It is important to note that if the OV is not 100% tumor-specific and is administered intravenously, then it can infect, not only the target tumor cells, but also some healthy normal cells in the tumor site. Even though intratumoral viral injections offer direct tumor infection, they are of limited use in regions (such as the brain) where the tumor cannot be reached directly [18]. Thus, intravenous virus administration would be the only viable option in those scenarios. Numerous pre-clinical attempts have been made to enhance the oncolytic potency of some oncolytic viruses, such as recombinant VSV vectors, with limited success.

Various mathematical models have been developed to investigate the dynamics of the oncolytic viruses on tumor cells [19–22]. None of the existing mathematical models, however, explicitly considers the effects of the potential adaptive immune responses against infected normal cells or against the virus itself after successful oncolytic virus propagation. For example, the mathematical models in [21, 22], describe the interactions of the immune cells with oncolytic viruses and tumor cells in virotherapy. While these two models incorporated the effects of adaptive immunity as the effector and memory immune responses, they did not consider tumor-immune interactions following successful onocolytic viral propagation. Additionally, these two models considered intratumoral injection of the oncolytic virus, while in our modeling attempt, we consider intravenous virus injection into the susceptible cell population.

Up to date, there is no mathematical model that delineates how oncolytic viruses that are not 100% tumor-specific can be used to augment oncolytic virotherapy with attenuated effects on normal cells. A recent study by Okamoto et al. [23] illustrates how infections of the normal cells by the oncolytic virus could enhance a cancer virotherapy prior to the accumulation of the adaptive immune response. They modeled how apparent competition between normal and tumor cell populations, both cell populations virally infected with a given oncolytic virus, can drive tumor cell population to extinction prior to accumulation of an adaptive immune response. While this model elucidated how infection of normal cells by oncolytic viruses can aid to increase the virus population size at the tumor site and reduce tumor burden, it did not take into account the fact that the oncolytic viral infection on the normal cells can induce unexpected and inevitable immune responses against the infected normal cell population.

Our proposed model also aims to elucidate the tumor-normal-immune-viral dynamics 1–4 days in the presence of immune response triggered by the escalated viral infection of normal cells. This is very important because the induction of activated CD8^+^ T cells into the tumor site may limit subsequent oncolytic virus spread and intratumoral infection. Even though we do not model the innate immune responses, it is important to note that the innate immune response against the virally-infected cells is often active in about 2–7 days post-infection [24].

## Materials and methods

### Mathematical model

**Fig 1:**
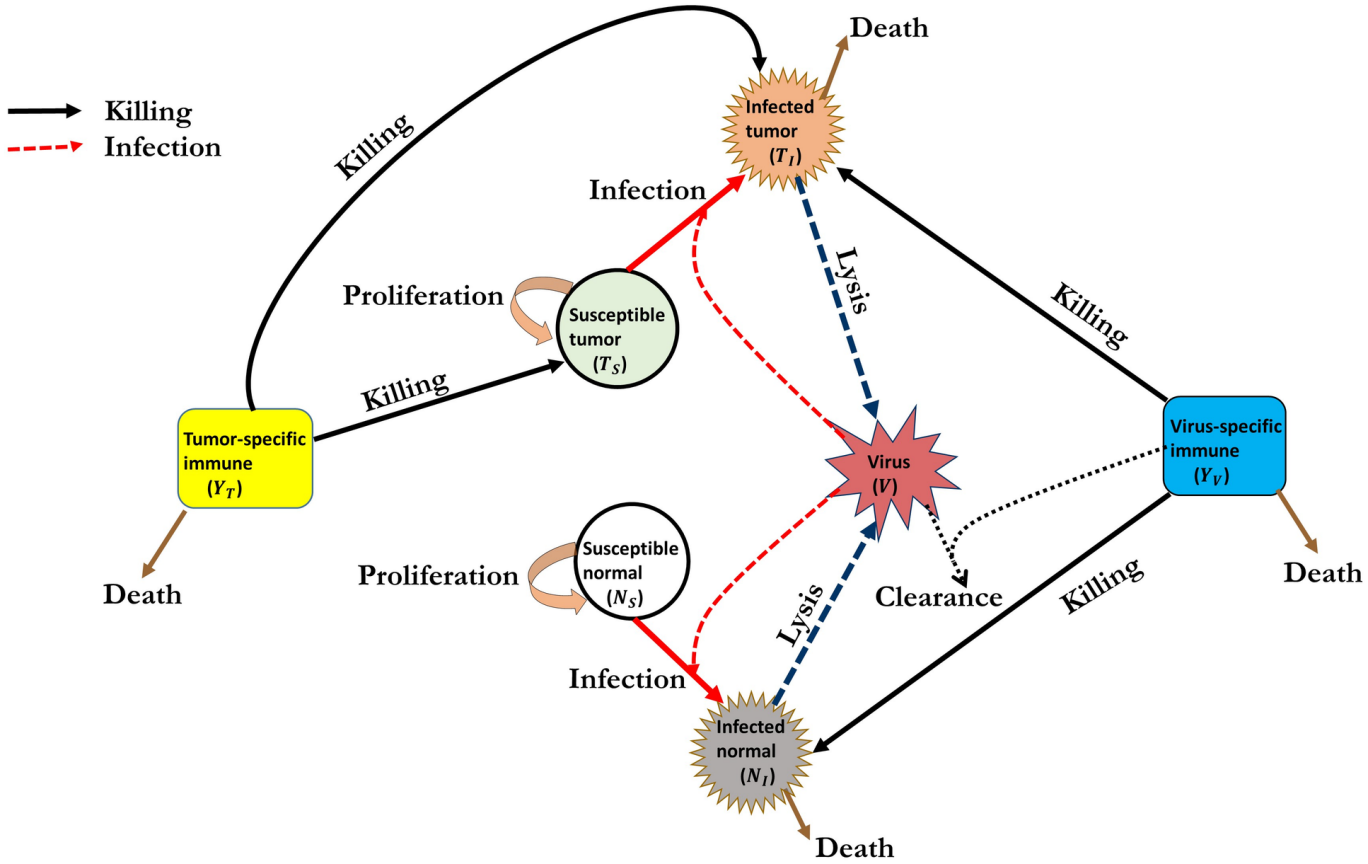
Interaction of immune cells and oncolytic virus with tumor cells. Susceptible (Uninfected) normal and tumor cells become infected by an oncolytic virus (vesicular stomatitis virus (VSV)). After successful viral propagation within the infected cells, infected cells undergo lysis (cell rupture) producing a progeny of new infectious viruses which spread and infect other susceptible cells. Debris from infected cells activates the virus-specific immune cells which then induces killing of infected cells and clearance of free virus. The tumor-specfic immune cells recognise (due to expression of tumor-associated antigens (TAAs)) and kill both uninfected and infected tumor cells.

The mathematical model is based on the diagram shown in Fig 1. The model describes the interactions between normal and tumor cells in the presence of the adaptive immune responses following an initial successful viral propagation phase on both normal and tumor cell populations. It consists of a system of delay differential equations (DDEs) with one discrete delay representing the time necessary to induce tumor-specific immune response. The main objectives of the proposed model are to predict: (1) the oncolytic viral tumor-specificity that maximises tumor reduction while minimizing the undesirable toxicity on normal tissue surrounding the tumor; (2) the effects of the potential antitumoral and antiviral immune responses in oncolytic virotherapy; and (3) tumor’s response to oncolytic viral infections, particularly, the model’s performance to single-viral and multi-viral injections strategies. For our modeling framework, we use the basic reproductive number *R*_0_ (see S1 Text in the Supporting Information) to indicate the combined therapeutic index of the oncolytic virus that is not 100% tumor-specific as a measure of oncolytic potency of the normal and tumor cell populations. Understanding the therapeutic index for oncolytic viruses is essential for the assessment of safety and selectivity of oncolytic viruses [25].

Our proposed model uniquely characterizes the impact of the oncolytic virus that is not 100% tumor-specific on the normal and tumor cell populations and further assesses the effects of corresponding antiviral and antitumoral adaptive immune responses following a successful virus propagation in oncolytic virotherapy. Here, the oncolytic virus that is not 100% tumor-specific is assumed to be a vesicular stomatitis virus (VSV), and the adaptive antitumor/antiviral immune cells are CD8^+^ T cells. We have chosen to use VSV in our model because it is capable of infecting a wide range of cell lines, has a genome that is easy to manipulate, and is capable of producing high viral titers [8, 31, 32]. More appropriate to our model, it has potential to infect both populations of normal and tumor cells. In order to allow the VSV to infect both normal and tumor cell populations, we assume that the viral injections into the system are administered intravenously and close to the tumor. One important assumption underlying our model is that the interaction kinetics between cell population and the VSV follow mass action kinetics, and all cell populations are homogeneously mixed as assumed in [33–35]. Homogeneous mixing implies that there are no different cell types within one cell population. Mass action kinetics are the appropriate interaction kinetics when one assumes that the density of the cell populations and viral particles is proportional to the total number of cells and viral particles [36]. Alternative to the mass action infection kinetics are the kinetics that account for the possibility of virus infection saturation at higher virus concentrations (e.g., see models in [37, 38]) or the virus infections that are frequency-dependent (e.g., see models in [20, 36]). Although such virus infection kinetics may be more realistic than mass action kinetics, they may, however, not be well known and may lead to more parameters.

The model’s variables are listed in Table 1.

**Table 1:** Model variables.

Variable	Description
*N*_*S*_(*t*)	the total number of susceptible (uninfected) normal cell population
*T*_*S*_(*t*)	the total number of susceptible (uninfected) tumor cell population
*N*_*I*_(*t*)	the total number of infected normal cell population
*T*_*I*_(*t*)	the total number infected tumor cell population
*V*(*t*)	the total number of oncolytic virions
*Y*_*T*_(*t*)	the total number of tumor-specific immune cells (primed tumor antigen-specific CD8^+^ T cells)
*Y*_*V*_(*t*)	the total number of virus-specific immune cells (primed antiviral CD8^+^ T cells)

### Model assumptions

The biological assumptions incorporated in the model based on the discussion above and the scientific literature are as follows:

1. The susceptible (uninfected) normal and tumor cells grow logistically at the rates, *r*_*N*_ and *r*_*T*_, up to their carrying capacities, *K*_*N*_ and *K*_*T*_, respectively. The choice of the logistic growth for uninfected tumor cells is based on the fact that tumors grow logistically in the absence of immune response [33]. Similarly, in the absence of cancer cells, normal cells are assumed to grow logistically [39].
2. For infected cell populations, we assume that their lifespan is much shorter than uninfected cell populations; hence, we do not need logistic growth.
3. Given that the oncolytic virus can successfully infect normal cells, we assume that normal cells, in the neighbourhood of tumor host tissue, can quickly self-renew during and after the oncolytic therapy [17].
4. To induce immune responses, oncolytic viruses are often designed to express immunostimulating cytokines, such as a granulocyte macrophage-colony stimulating factor [GM-CSF] [40] and interleukin [IL]-2 [41]. We, therefore, assume that oncolytic virus infection on both normal and tumor cell populations can induce virus-specific immune responses mediated by antiviral CD8^+^ T cells [42].
5. We assume that tumor-specific immune cells (antitumor CD8^+^ T cells) can recognise and kill both uninfected and infected tumor cells because tumors often express tumor-associated antigens (TAAs) [43, 44].
6. We assume that there is no virus-specific immunity prior to oncolytic virotherapy, and hence all infected cell populations, and virus-specific immune cells start at size 0. On the other hand, we assume that the initial size of the susceptible (uninfected) normal and tumor cell populations is equivalent to the size determined by the experiments at time 0 of tumor detection. Thus, we assume that tumor-specific immunity, measured by the number of antitumor CD8^+^ T cells at the tumor site, exists at the start of oncolytic virotherapy.
7. We also assume that upon lysis of an infected cell, a progeny of new infectious oncolytic viruses bursts out of the lysed cell, and infect neighbouring uninfected cells.

### Model equations

The model consists of the following delay differential equations (DDEs):

$$\begin{array}{cc} \frac{dN_{S}}{dt} & =\underset{\text{proliferation}}{\underset{︸}{r_{N}N_{S}(1-\frac{N_{S}+N_{I}}{K_{N}})}}-\underset{\text{infection}}{\underset{︸}{\beta_{N}N_{S}V}} \end{array}$$ (1)

$$\begin{array}{cc} \frac{dT_{S}}{dt} & =\underset{\text{proliferation}}{\underset{︸}{r_{T}T_{S}(1-\frac{T_{S}+T_{I}}{K_{T}})}}-\underset{\text{infection}}{\underset{︸}{\beta_{T}T_{S}V}}-\underset{\text{killing}\;\text{by}\;\text{immune}\;\text{cells}}{\underset{︸}{\gamma_{T}\frac{Y_{T}}{h_{Y}+Y_{T}}T_{S}}} \end{array}$$ (2)

$$\begin{array}{cc} \frac{dN_{I}}{dt} & =\underset{\text{infection}}{\underset{︸}{\beta_{N}N_{S}V}}-\underset{\text{lysis}}{\underset{︸}{\lambda_{N}N_{I}}}-\underset{\text{killing}\;\text{by}\;\text{immune}\;\text{cells}}{\underset{︸}{\gamma_{V}Y_{V}N_{I}}} \end{array}$$ (3)

$$\begin{array}{cc} \frac{dT_{I}}{dt} & =\underset{\text{infection}}{\underset{︸}{\beta_{T}T_{S}V}}-\underset{\text{lysis}}{\underset{︸}{\lambda_{T}T_{I}}}-\underset{\text{killing}\;\text{by}\;\text{immune}\;\text{cells}}{\underset{︸}{\gamma_{T}\frac{Y_{T}}{h_{Y}+Y_{T}}T_{I}}}-\underset{\text{killing}\;\text{by}\;\text{immune}\;\text{cells}}{\underset{︸}{\gamma_{V}Y_{V}T_{I}}} \end{array}$$ (4)

$$\begin{array}{cc} \frac{dV}{dt} & =\underset{\text{lysis}}{\underset{︸}{b_{T}\lambda_{T}T_{I}}}+\underset{\text{lysis}}{\underset{︸}{b_{N}\lambda_{N}N_{I}}}-\underset{\text{clearance}}{\underset{︸}{\omega V}} \end{array}$$ (5)

$$\begin{array}{cc} \frac{dY_{T}}{dt} & =\underset{\text{recruitment}}{\underset{︸}{p_{T}\frac{T_{S}+T_{I}}{h_{T}+T_{S}+T_{I}}}}-\underset{\text{death}}{\underset{︸}{\delta_{T}Y_{T}}} \end{array}$$ (6)

$$\begin{array}{cc} \frac{dY_{V}}{dt} & =\underset{\text{recruitment}}{\underset{︸}{p_{V}(T_{I}\left(t-\tau \right)+N_{I}\left(t-\tau \right))}}-\underset{\text{death}}{\underset{︸}{\delta_{V}Y_{V}}} \end{array}$$ (7)

The initial conditions of the model are as follows at *t* = 0: *N*_*S*_ = 10^11^ cells; *T*_*S*_ = 10^6^ cells; *N*_*I*_ = 0 cells; *T*_*I*_ = 0 cells; *Y*_*T*_ = *Y*_*V*_ = 0 cells; *V*(*t*) = 10^9^ plaque-forming units (PFU). PFU is a globally accepted measurement for infectious titers (virus particles); non-infectious (defective) virions that are incapable of forming plaques cannot infect their target cells, and thus are excluded when counting the plaque-forming units. For *τ* ≤ *t* ≤ 0, we have constant history functions of cell concentrations on that time interval. Thus, we implicitly assume that the system was at equilibrium prior to time 0 and apply to above conditions at *t* = 0.

**In** Eq 1, the first term, $r_{N}N_{S}(1-\frac{N_{S}+N_{I}}{K_{N}})$, represents a logistic growth of the normal cells with an intrinsic growth rate *r*_*N*_ and the carrying capacity *K*_*N*_. Note, the normal cell population consists of uninfected (*N*_*S*_) and infected cells (*N*_*I*_). Since the uninfected normal cells can become infected with the oncolytic virus at the rate *β*_*N*_, the second term, −*β*_*N*_*N*_*S*_*V*, denotes the reduction of normal cell population due infection with the oncolytic virus.

**In** Eq 2, the logistic tumor cell growth of the uninfected tumor cells is denoted by the term, $r_{T}T_{S}(1-\frac{T_{S}+T_{I}}{K_{T}})$ with the intrinsic growth rate *r*_*T*_ and the carrying capacity *K*_*T*_. Similarly, during oncolytic virotherapy, the tumor cell population is sub-divided into two sub-populations, the uninfected cells represented by *T*_*S*_ and infected tumor cells denoted by *T*_*I*_. The uninfected tumor cells become infected by the oncolytic virus at the rate *β*_*T*_. Hence, the second term, −*β*_*T*_*T*_*S*_*V*, represents the reduction of the tumor cell population as a result of a successful viral oncolysis (i.e., viral replication and burst). Since some oncolytic viruses, such as the vesicular stomatitis virus (VSV), are capable of inducing the antitumor immune response against the infected tumor cells [31, 45], the third term, $-\gamma_{T}\frac{Y_{T}}{h_{Y}+Y_{T}}T_{S}$, represents the reduction of the tumor cell population by the antitumor adaptive immune response. The interaction between tumor and the tumor-specific immune cells follows the Michaelis-Menten kinetics because immune cell infiltration into the tumor is often restricted by tumor architecture [46]. Thus, *γ*_*T*_ denotes the rate at which tumor cells are lysed by the tumor-specific immune cells and *h*_*Y*_ represents the half-saturation constant of immune cells that supports half the maximum killing rate.

**In** Eq 3, the first term, *β*_*N*_*N*_*S*_*V*, represents the number of normal cells that become infected with the oncolytic virus. The second term, −*λ*_*N*_*N*_*I*_, denotes the death of the infected normal cells at the rate *λ*_*N*_. Experimental evidence indicates that death of infected normal cells may be attributed to apoptosis of the infected cells in attempt to inhibit virus propagation [47]. Therefore, we assume that the infection by the oncolytic virus also induces the adaptive antiviral immune response to infected cells [48]. The third term, −*γ*_*V*_*Y*_*V*_*N*_*I*_, represents the number of infected normal cells lysed by the antiviral immunity with lysis rate *γ*_*V*_.

**In** Eq 4, the first term, *β*_*T*_*T*_*S*_*V*, represents the number of tumor cells that become infected with the virus. The second term, −*λ*_*T*_*T*_*I*_, denotes the death of the infected tumor cells at the OV-induced death rate *λ*_*T*_. Again, since tumor architecture may hinder the adaptive antitumor immune cell infiltration [46], we consider the Michaelis-Menten kinetics for the interaction between infected tumor cells and the adaptive antitumor immune response. Hence, we model this scenario with the term $\gamma_{T}\frac{Y_{T}}{h_{Y}+Y_{T}}T_{I}$. The last term, −*γ*_*V*_*Y*_*V*_*T*_*I*_, represents the number of infected tumor cells that become lysed by the virus-specific immune cells.

**In** Eq 5, upon successful viral infection and replication, the infected tumor cells die and new oncolytic virus particles that are released from the infected tumor cell. Thus, *b*_*T*_ is the burst size for viruses from an infected tumor cell. The first term, *b*_*T*_*λ*_*T*_*T*_*I*_, represents the production of new oncolytic virus particles released from infected tumor cells after a successful viral propagation. Similarly, the second term, *b*_*N*_*λ*_*N*_*N*_*I*_, denotes the production of new viral particles released from the successful oncolysis of the infected normal cells. Here, *b*_*N*_ is the burst size for viruses from an infected normal cell. Finally, the last term, *ωV*, denotes the viral clearance of the free virus from the host body by virus-specific immune cells, at the clearance a rate *ω*.

**In** Eq 6, the adaptive antitumor immune response depends of the cross-priming of the T-cells by mature antigen presenting cells (e.g. macrophages) with the antigens expressed on both infected and uninfected tumor cells [49, 50]. For simplicity, we assume that such a priming process has been successful and we do not model the kinetics of priming, instead we incorporate delay terms of immune response to viral infections. The first term, $p_{T}\frac{T_{S}+T_{I}}{h_{T}+T_{S}+T_{I}}$, represents the antitumoral immune response against the tumor cells, with the immune cell recruitment rate *p*_*T*_. Since activation of antitumoral immune response, mediated by CD8^+^ T cells, is dependent on the amount of tumor antigens, we use Michaelis-Menten term to indicate the saturation effects of the tumor-specfic immune response [29, 51]. For simplicity, we use the same half-saturation constant of tumor antigens that induce half proliferation of immune cells, *h*_*Y*_, as the half-saturation constant of adaptive immune cells that supports half the maximum killing rate (to both viral- and tumor-specific CD8^+^ T cells), *h*_*T*_. Finally, the last term, −*δ*_*T*_*Y*_*T*_, denotes that the adaptive tumor-specific immune population declines as a result of natural cell death, at the intrinsic death rate *δ*_*T*_.

**In** Eq 7, a delayed immune response to virus infection to both normal and tumor cells is modeled by the term *p*_*V*_(*T*_*I*_(*t* − *τ*) + *N*_*I*_(*t* − *τ*)), where a parameter *p*_*V*_ is a virus-specific proliferate rate of the antiviral immune cells due to the presence of virus particles (virus antigens) on the surface of the infected cells. Immune response to viral antigens require time necessary for cell activation and proliferation. That means, antigenic stimulation generating the antiviral immune response, mediated by T cells, require a period of time *τ*, which may depend on prior antigenic stimulation period *t* − *τ*. Note that the delay of antiviral immune response is also crucial for enabling first round of oncolytic virus replication and subsequent release of the viral progeny [48]. The last term, −*δ*_*V*_*Y*_*V*_, represents the natural death, with the death rate −*δ*_*V*_, of the adaptive virus-specific immune cells.

#### Parameter estimation

Known parameter values were taken from available literature, while unknown parameters were estimated based on the rational biological information of such parameters. Please refer to S1 Text in the Supporting Information for details about parameter estimation. The baseline parameters used in the model simulations are given in Table 2.

**Table 2:** Parameter values used in the model simulations.

Parameter	Description	Value	Source
*r*_*N*_	the intrinsic growth rate of normal cells	0.00275 hr^−1^	[23]
*K*_*N*_	the carrying capacity of normal cells	10^11^ cells	[23]
*β*_*N*_	the rate at which VSV infects normal cells	(1.7 × 10^−8^)/24 virion^−1^ hr^−1^	rescaled from daily rate in Friedman et al. [26]
*r*_*T*_	the intrinsic growth rate of tumor cells	0.003 hr^−1^	[23, 27]
*K*_*T*_	the carrying capacity of tumor cells	1.47 × 10^12^ cells	[23]
*β*_*T*_	the rate at which VSV infects tumor cells	(5 × 10^−12.5^, 5 × 10^14^) virion^−1^ hr^−1^	[23]
*γ*_*T*_	lysis rate of susceptible tumor cells by tumor-specific immune cells	1/24 hr^−1^	rescaled from daily rate in Eftimie et al. [22]
*h*_*Y*_	the half-saturation constant of immune cells	40 cells	[22]
*λ*_*N*_	the death rate of infected normal cells	1/24 cells hr^−1^	estimate
*γ*_*V*_	lysis rate of the infected normal cells by virus-specific immune cells	1/24 cells^−1^ hr^−1^	estimate
*λ*_*T*_	death rate of infected tumor cells due to VSV lysis	1/24 cells^−1^ hr^−1^	rescaled from daily rate in Eftimie et al. [22]
*b*_*T*_	the burst size from tumor cells lysed by VSV	1350 virions cell^−1^	[23]
*b*_*N*_	the burst size from normal cells lysed by VSV	1000 virions cell^−1^	Estimate
*ω*	virus clearance rate	2.5 × 10^−2^ hr^−1^	[26, 28]
*p*_*V*_	proliferation rate of virus-specific immune cells in response to VSV antigens	0.025−0.1042 hr^−1^	rescaled from daily rate in Eftimie et al. [22]
*δ*_*V*_	death rate of the virus-specific immune cells	5.54 × 10^−3^ hr^−1^	rescaled from daily rate in Eftimie et al. [22]
*p*_*T*_	proliferation rate of tumor-specific immune cells	0.0375/24 hr^−1^	rescaled from daily
*h*_*T*_	the half-saturation constant of tumor cells in response to tumor antigens	40 cells	[22] rate in de Pillis et al. [29]
*δ*_*T*_	death rate of the tumor-specific immune cells	3.75 × 10^−4^ hr^−1^	rescaled from daily rate in de Pillis et al. [30]

### Model analysis

To better understand the dynamics of the proposed model, we begin by examining the model’s behavior about the steady states in the absence of the virus. This analysis is crucial for identifying the parameters of the model that help to achieve a tumour-free state without oncolytic virotherapy. Additionally, this analysis would be important for comprehending the effect of the adaptive immune response following oncolytic virotherapy. In S1 Text, we derive the model’s basic reproductive number, *R*_0_, and provide an analysis of the model’s virus free equilibrium points and determine their stability in terms of *R*_0_, respectively. The non-trivial steady states of the model without virus (i.e., *N*_*I*_ = *T*_*I*_ = *Y*_*V*_ = *V* = 0) are found by equating Eqs (1–7) to zero, which results in the following virus free steady states:

$$E_{N}≔(K_{N},0,0,0,0,0,0)\;\;\;\text{Tumor-free}\;\text{(TF)}\;\text{steady}\;\text{state},$$ (8)

$$\begin{array}{c} E_{T}≔(0,0,T_{S0},0,0,0,Y_{T0})\;\;\;\text{Tumor-only}\;\text{(TO)}\;\text{steady}\;\text{state}\\ \;\;\text{(i.e.,}\;\text{tumor}\;\text{without}\;\text{the}\;\text{surrounding}\;\text{normal}\;\text{cells)}, \end{array}$$ (9)

$$\begin{array}{c} E_{NT}≔(K_{N},0,T_{S0},0,0,0,Y_{T0})\;\;\;\text{Co-existence}\;\text{steady}\;\text{state}\\ \;\text{(i.e.,}\;\text{tumor}\;\text{and}\;\text{the}\;\text{surrounding}\;\text{normal}\;\text{cells}\;\text{are}\;\text{present)}, \end{array}$$ (10)

where

$$\begin{array}{c} \begin{array}{c} T_{S0}≔\frac{-b+\sqrt{b^{2}-4ac}}{2a},\;\;\;\text{and}\;\;\;Y_{T0}=\frac{p_{T}T_{S0}}{\delta_{T}(T_{S0}+h_{T})} \end{array} \end{array}$$

with

$$\begin{array}{c} \begin{array}{c} a≔r_{T}\xi ,\;\;\;\xi =h_{Y}\delta_{T}+p_{T}\\ b≔(\gamma_{T}-r_{T})K_{T}\xi +(h_{T}r_{T}-K_{T}\gamma_{T})h_{Y}\delta_{T}\\ c≔-K_{T}\delta_{T}h_{T}h_{Y}r_{T}. \end{array} \end{array}$$

The detailed mathematical proofs of the stability analyses associated with these steady states are provided in S1 Text. In particular, we derive the basic reproductive number, *R*_0_, which is a measure of the infection on the populations of normal and tumor cells with the oncolytic virus (see S1 Text in the Supporting Information for a detailed discussion on how to calculate *R*_0_), of the model given by

$$\begin{array}{c} R_{0}=R_{0N}+R_{0T} \end{array}$$ (11)

where

i $R_{0N}≔\frac{b_{N}\beta_{N}N_{S}}{\omega },$ represents the basic reproductive number of the virus when introduced into a population of normal cells only.
ii $R_{0T}≔\frac{(Y_{T}+h_{T})b_{T}\beta_{T}\lambda_{T}T_{S}}{((Y_{T}+h_{T})\lambda_{T}+Y_{T}\gamma_{T})\omega },$ represents the basic reproductive number of the virus when introduced into a population of tumor cells only.

A fundamental result about the equilibria analysis of the system described by the Eqs 1–7 is given by the following proposition:

**Proposition 1.** *The virus free equilibrium points* *E*_*N*_ *and* *E*_*T*_ *are always unstable, while* *E*_*NT*_ *is locally asymptotically stable if and only if* *R*_0_ < 1.

Refer to S1 Text in the Supporting Information for the detailed proofs corresponding to the above proposition.

## Results

### Numerical simulations

The numerical solutions of our model eqs (1–7) along with the initial conditions were carried out using MATLAB *dde23*. The generic MATLAB source code used to calculate the model solutions is provided in S1 Text in the Supporting Information. We first investigate the system’s long-term behavior. Note, at time *t* < 7, we assume that there are no virus-specific immune cells at the tumor site in order to allow the virus to infect, replicate and kill some infected cells. For all the simulations, we assumed that the susceptible tumor begins at the size measured at time *t* = 0 hours in an immunocompetent host. In experiments, tumor size is often measured in volume (*mm*^3^), then in our model we convert tumor volume to cell population by assuming that 1 *mm*^3^ ≈ 1 × 10^6^ tumor cells, as has been done in [20, 34].

#### Comparing with previous studies

**Fig 2:**
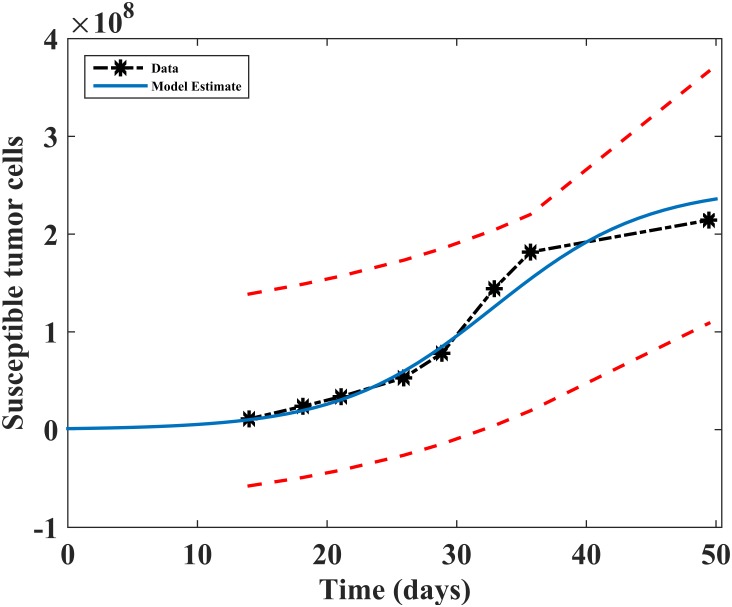
Model fit to uninfected tumor growth data. Model fitting to experimental tumor growth data using Eq 2, the uninfected (susceptible) tumor cell population, *T*_*S*_, and other model variables set to zero. The susceptible tumor cell population is fitted to the data with two-sided 95% confidence intervals (dashed lines) computed from exponential distribution statistics. A black dashed line is just a straight line between data points. Parameter values are *r*_*T*_ = 0.00258, *K*_*T*_ = 3.12 × 10^8^, *β*_*T*_ = *γ*_*T*_ = 0.

To facilitate comparison of our model findings with other mathematical models, in particular with the model by Okamoto et al. [23], we present numerical simulations where the therapeutic dose is *V* = 10^9^ pfu of the initial free virus load. As a first step in evaluating the performance and accuracy of a model in predicting tumor growth, we fit our model to the available experimental tumor data used in the model by Bajzer et al. [52], who obtained it from the in vivo experiments of human myeloma tumor xenografts implanted in immunodeficient mice [53]. The data in [52, 53] reports both the untreated and treated (when virotherapy was introduced on day 15 after the implantation of multiple myeloma xenografts in mice) tumor growth. We used the untreated tumor growth data to estimate the daily tumor growth rate (*r*_*T*_) by fitting a sub-form of our model and evaluated the accuracy of the numerical simulations. The fitting of the sub-form of our model was done by minimizing the sum of square errors (SSE) between the experimental data points and the model output using the MATLAB function *lsqnonlin*. Our model fit, with a 95% confidence interval, is shown in Fig 2. Since one of the goals of our study is to predict tumor’s response to oncolytic viral infection, we observe from Fig 2 that the model fits (with the susceptible cell population taken as the baseline variable) the tumor growth data fairly well. This observation provides some assurance that uncertain model parameters fall within 95% confidence of the true tumor growth. We further assessed our model parameter sensitivity through a global sensitivity analysis in the subsequent section in order to gain a better understanding of the model’s behavior to small variations in the parameters.

#### Global sensitivity analysis (GSA)

For our model, we performed the GSA because a large number of parameters. Most important, we perform sensitivity analysis in order to identify key parameters that can be varied to achieve plausible oncolytic potency and reduced tumor-specificity of the oncolytic virus that is not 100% tumor-specific in oncolytic Virotherapy. Following the numerical method described in [54], we per- formed Latin hypercube sampling. We generated 1000 samples to compute the partial rank correlation coefficients (PRCC) and the associated p-values with respect to virus infection at 24-hour intervals up to 96 hours. The sensitivity indices of the PRCC, ranging from −1 to +1, indicate the strength of the monotonic relation between the susceptible cell population and parameter of interest. A PRCC index of −1 indicates a strong negative monotonic relationship between a given parameter and the model variable(s) (i.e., susceptible normal and tumor cells in this case), while the index of +1 shows a strong positive monotonic relationship between the given parameter and model variable.

#### The GSA results: Model implications for oncolytic virotherapy

**Fig 3:**
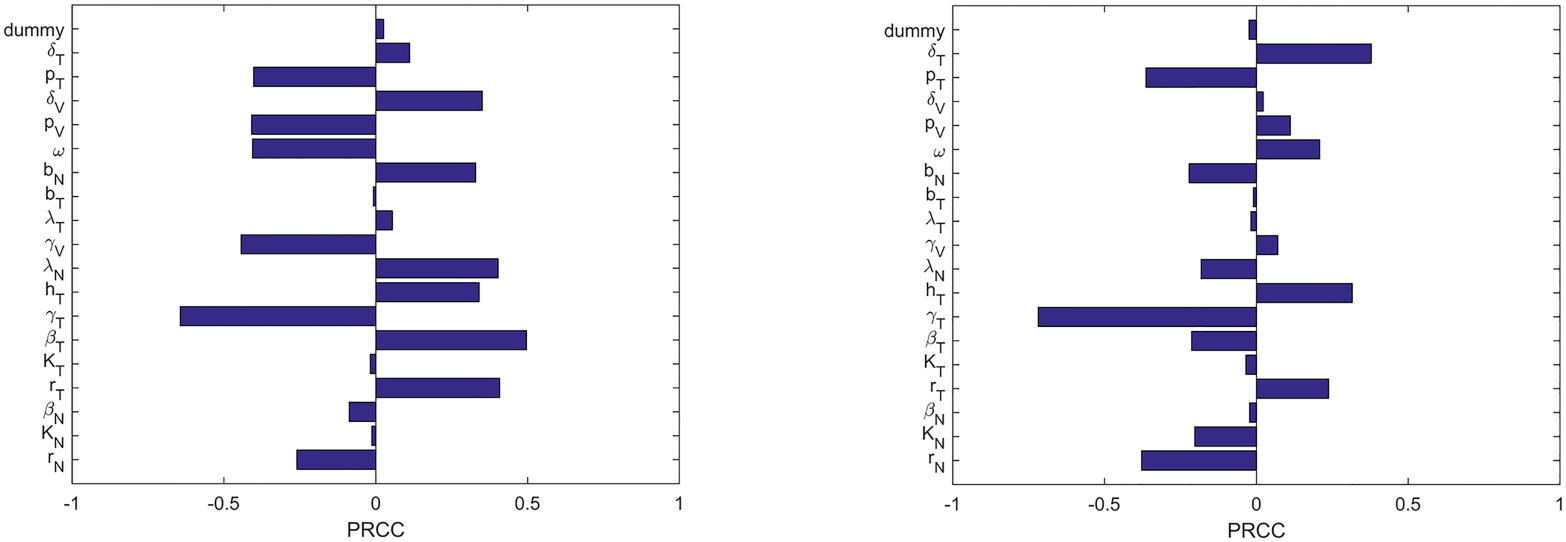
Snapshots of the sensitivity analysis of the model. Sensitivity indices of the model parameters with oncolytic virus taken as a baseline PRCC analysis variable. Analysis was computed based on the baseline parameter values presented in Table 2, with a viral dose of *V* = 10^9^ plaque-forming units (pfu). The sensitivity analysis shows statistically significant PRCC values (p-value < 0.01) at: (a) 24 hours and (b) 96 hours, respectively.

We investigated the parameter sensitivity analysis with *τ* = 0 in the eqs 1–7. Sensitivity analysis of our model without delay (i.e., when the parameter *τ* = 0), Eqs 1–7. We present only two time snapshots in Fig 3.

Fig 3 reveals high sensitivity of the model to small parameter changes at 24 and 96 hours. Most important, this global sensitivity analysis indicates which parameters account for the most variation in the early or late stages of oncolytic virotherapy. From a treatment perspective, this is essential for identifying which parameters of the model could be the “key drivers” of the success of the virotherapy at any time point. In Fig 3, note that the PRCC algorithm usually assigns a PRCC value to the control variable named “dummy”. This dummy parameter is not part of the model parameters, and hence, it does not affect the model results in any way. According to the PRCC algorithm, the model parameters with sensitivity index less than or equal to that of the dummy parameter are usually taken to be not significantly different from zero (with p-value > 0.01) [54].

The PRCC subplots in Fig 3, correspond to the times of giving the single-viral dose of *V* = 10^9^ virions at the 24 and 96 hours with the initial dose given at 24-hours after the start of the tumor treatment. At time *t* = 24 hours, Fig 3(a) indicates that a number of parameters are statistically different from zero (with p-value < 0.01) The significant parameters include: the rate of VSV infection to tumor cells, *β*_*T*_, half saturation constant of tumor infected cells, *h*_*T*_, the death rate of infected normal cells, *λ*_*N*_, death rate of the virus-specific immune cells, *δ*_*V*_, the proliferation rate of tumor-specific immune cells *p*_*T*_, and the lysis rate of the infected normal cells by virus-specific immune cells (*γ*_*V*_).

From the treatment perspective, the result of the sensitivity analysis shows that infection of normal cells can induce an antiviral immune response that could quickly eliminate the infected cells. This suggests that oncolytic viral infection of normal cells can be useful only when the virus replicates rapidly within infected normal cells. At *t* = 96 hours, the intrinsic growth rates, *r*_*N*_ and *r*_*T*_, of normal and tumor cells also become consistently influential on normal and tumor cell populations, respectively. Similarly, death rate of the tumor-specific immune cells, *δ*_*T*_, proliferation rate of tumor-specific immune cells, *p*_*T*_, and the half-saturation constant of the adaptive immune cells, *h*_*T*_, also become statistically significant at later time point (i.e., time *t* = 96 hours).

Based on this global sensitivity analysis, we deduce at following treatment implications: (i) For a period of less than 4 days, apart from direct oncolysis, an oncolytic therapy should target recruiting more tumor-specific cells to augment the therapy. This could be achieved by engineering the VSV to express a tumor antigen directly [55]. Viral infections usually trigger an immune response that is essential for elimination of tumor cells [6, 47]. This sensitivity analysis indicates why it is currently not easy to treat tumors within 4 days with oncolytic viruses, from on the onset of tumorigenesis. The precise time of oncogenesis in clinic is very difficult to determine. (ii) When designing the oncolytic viruses, such as vesicular stomatitis virus (VSV) [7] or Newcastle disease virus (NDV) [56], that are not 100% tumor-specific, it is important that such viruses replicate rapidly within normal cells since normal cells can quickly become more sensitive and inhibitory to virus replication over time. Global sensitivity analysis illustrates that the model is less sensitive to early viral infection (see Fig 3(a)) on the normal cell population, and becomes increasingly sensitive at later time point (Fig 3(b)). (iii) Tumor aggressiveness as well as the strength of the patient tumor-specific immunity may predict patient response to oncolytic virotherapy.

### Treatment strategies

Having determined which parameters are most influential in our model, we now investigate two main dosing treatment strategies in oncolytic virotherapy: (1) single-viral dose (i.e., one viral dose administered at three different time points once), and (2) periodic dosing (i.e., one viral dose given at three successive time points). Currently, a full understanding of the best plausible protocols to administer oncolytic viruses to cancer patients is still very limited. This is partly because there are no precise clinical results for comparing two different oncolytic virotherapeutics administered through identical routes in the same types of tumor. It is important to note that a comprehensive comparison of clinical virotherapy trial regimens is time-consuming and complicated [57]. There is still no common consensus regarding:

(i) the oncolytic virus dosages (i.e., low versus high dosage. The optimum oncolytic virus dosage in the clinic is still unknown [58]; although virus inoculum is often manipulated in clinical trials in orders of magnitude (10^3^−10^10^) pfu [59]),
(ii) the appropriate dosing intervals (i.e., oncolytic virus repetitive times: hourly, weekly or monthly. A detailed review of clinical dosing intervals of various oncolytic viruses is reported in [57]),
(iii) the best virus delivery route (e.g., systemic delivery versus intratumoral delivery. Recent clinical application of oncolytic viruses in these routes is reviewed in [57, 60]),
(iv) the virus administration scheme (i.e., single- versus multiple-dose [61]. The appropriate dosing schedule of oncolytic viruses in the clinic is still not precisely defined [58]).

Although some of the above issues have explored in several studies (e.g., see reviews in [62, 63]), in the present study we address some of these challenges, in particular (ii) and (iv), from the quantitative point of view that involves the basic reproductive number, *R*_0_, of the model. In the subsequent section, we provide brief guidelines underlining the use of *R*_0_ analysis that conforms with plausible biological outcomes of our model. Most importantly, *R*_0_ analysis, along with model simulations, would help to understand the qualitative behavior of the virus dynamics in our model, identify essential parameters necessary for tumor extinction or at least a controlled tumor state, and suggest possible future directions for further oncolytic virotherapy research.

#### Oncolytic viral infection dynamics

When designing an oncolytic virus, some important considerations include administration of variety of dosing schemes and testing different viral doses to ensure clinical safety [64]. Here, we present the results of the model with respect to the free-virus steady state as described in S1 Text as our initial steady state. In this steady state, it is interesting to investigate the effects of the virotherapy because:

1. When the basic reproductive number of the model is less than one (*R*_0_ < 1), then the oncolytic virus uses both normal and tumor cell populations for its replication. Note that, in general, if *R*_0_ > 1, the viral infections will continue to spread in at least one cell population, as implied by the case **(i.)** in S1 Text. It is essential to note that if *R*_0_ < 1, viral infections will eventually disappear from both tumor and normal cell populations over time, as implied by the case **(ii.)** in S1 Text. Of particular interest, we note and define the following conditions on *R*_0*N*_:
  (i) If *R*_0*N*_ < 1, then the infection on normal cell population will ultimately vanish over time.
  (ii) When designing an oncolytic virus that is not 100% tumor-specific, it is important to ensure that the basic reproductive number of normal cells, *R*_0*N*_, is less than that of tumor cells, *R*_0*T*_. In this case, the virus would infect more tumor cells than normal cells as evidenced by a large progeny virions from dead tumor cells [65].
  (iii) $R_{0N}<R_{0N}^{⋆}$, where $R_{0N}^{⋆}$ is the maximum value of the basic reproductive number for the normal cell population.
  (iv) Evidence suggests that the number of new virions produced from infected normal cells is somehow proportional to that produced from tumor cells [56]. Thus, we take *R*_0*N*_ = *α*(1 − *R*_0*T*_), where *α* is a constant fraction, as explained in **Brief guidelines for *R*_0_ analysis** in S1 Text.
2. In the case where *R*_0_ < 1, the viral infection on normal cells would invoke the immune response (T-cells or NK-cells) which may eliminate the virus-infected cells [66]. Thus, the infection of normal cells has two therapeutic outcomes:
  (i) If the virus replicates and lyses infected cells quickly, then oncolytic therapy may be enhanced by production of new virions, which can then spread to uninfected tumor cells. Evidence indicates that fast replicating viruses (i.e., those that can lyse infected cells quickly), can avoid being engulfed by innate and adaptive immune cells, and have a greater opportunity to further infect uninfected cells [67].
  (ii) Early removal of infected cells might inhibit success of the oncolytic therapy [19], but late immune response involvement might be necessary for clearing both infected normal and tumor (both uninfected and infected) cells [68].
3. When the basic reproductive number of the model is greater than one (*R*_0_ > 1), then the oncolytic virus endemically uses either normal or tumor cell population for its replication. From the treatment point of view, having *R*_0_ > 1 is an undesirable treatment result in virotherapy because *R*_0_ > 1, implies that viral infections will continue to spread in at least on cell population. Note that the basic reproductive number of the model, *R*_0_ (see S1 Text in the Supporting Information), is composed of two basic reproductive numbers, *R*_0*N*_ and *R*_0*T*_, of normal and tumor cells, respectively. When at least one of the basic reproductive numbers is greater than unity, then the cell population corresponding to the one with the basic reproductive number greater than one would be the one in which viral infection will persists forever. Further investigation of this condition (i.e., when *R*_0_ > 1) constitutes one of the possible model extensions which will be incorporated in the future work.

#### Experimental dosing scenarios

**Fig 4:**
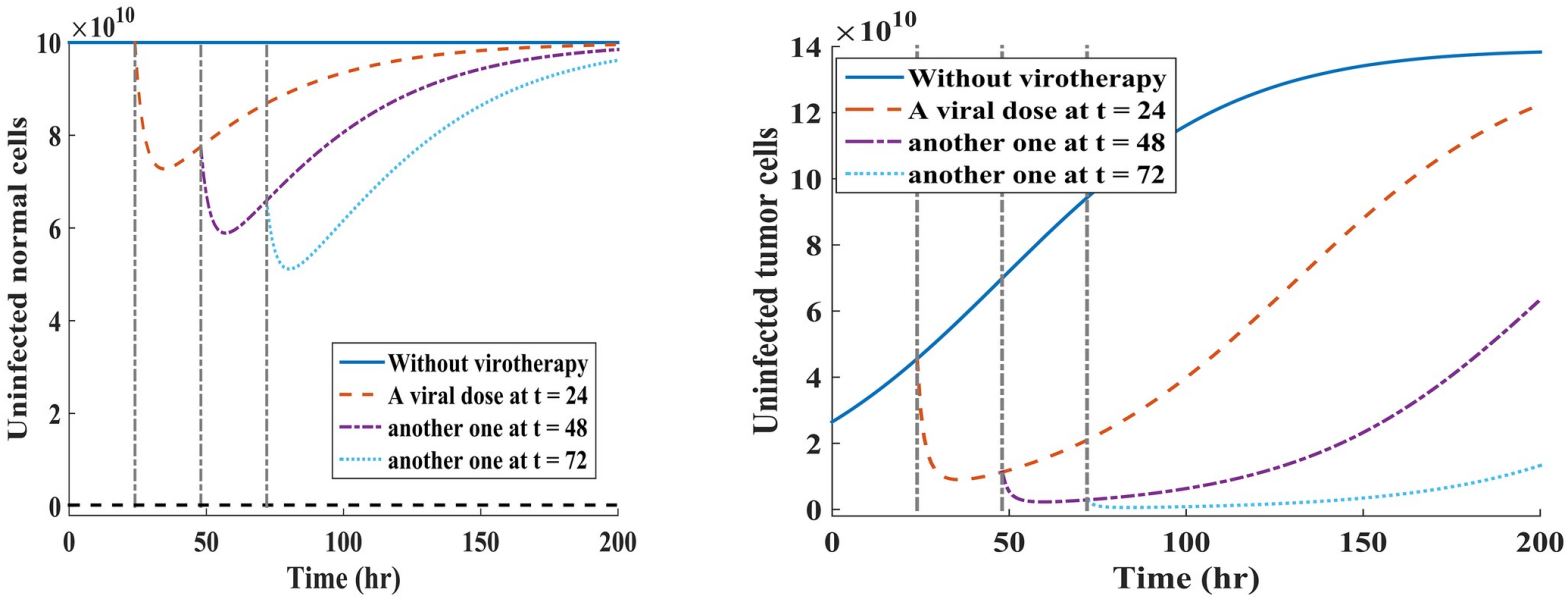
Multi-viral dosing scheme under scenario 1. Plots of the susceptible normal and tumor cell populations when a virus is administered at three successive times, with a viral dose of *V* = 10^9^ pfu when $R_{0N}=\frac{\left(1-R_{0T}\right)}{2}$. Fig 4(a) shows how the oncolytic virus reduces the susceptible normal cell population during multiple-viral dose scheme. Fig 4(b) shows how successive viral doses can lead to tumor eradication or at least keep the tumor in transient dormancy, which is followed by tumor relapse.

**Fig 5:**
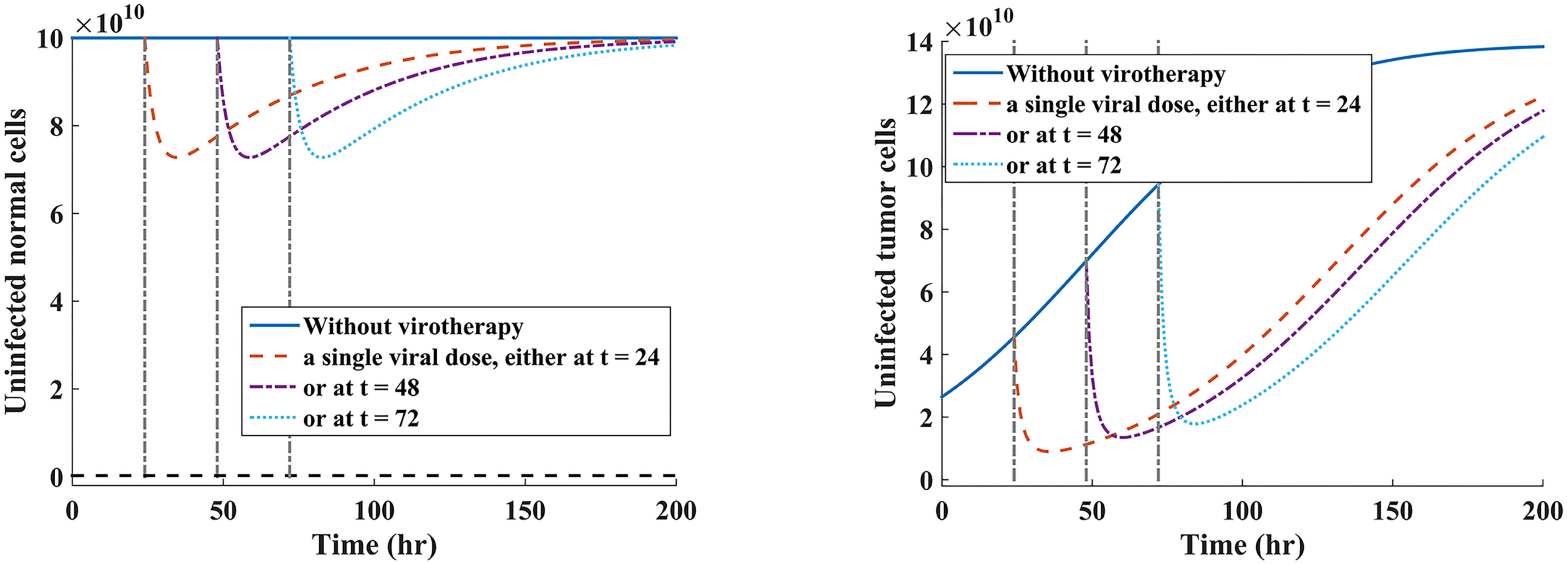
Single-viral dosing scheme under scenario 2. Plots of individual susceptible normal and tumor cell populations when the single dose of *V* = 10^9^ pfu is administered at three different time points when $R_{0N}=\frac{\left(1-R_{0T}\right)}{2}$. Fig 5(a) shows a reduction and rapid self-renewing of the susceptible normal cell population during an oncolytic virotherapy. Fig 5(b) shows the reduction of uninfected tumor cell population under the single-viral dose scheme.

Here we examine the hypothetical clinical dosing schedule of the oncolytic virus to test whether this would yield better treatment response of our hypothetical patient under single-viral dose (Scenario 1) and periodic dosing (Scenario 2). The rationale behind comparison of these treatment strategies in our model is motivated by (ii) and (iv) in **Treatment strategies**. We are interested in investigating virus dynamics for hourly dosing intervals under the two virus administration scenarios (i.e., single- versus multiple-dose). We kept the same viral dosage regimen of *V* = 10^9^ pfu for all treatment scenarios. Maintaining the same virus injection dosage is often done in experimental research (e.g., see [69, 70]). Note that for all the simulations, the value of *τ* is fixed at 7 hours, and *τ* was shown not to affect the stability of the virus-free equilibrium points. Hence, we have omitted the effects of time-varying delays in the present discussions. The results of the model simulations when *R*_0_ < 1 are given in Fig 4 for periodic dosing scenario, and Fig 5 for single-viral dose scenario.

Given that the infected normal tissues in the neighbourhood of the tumor has capacity to self-regenerate [17], we note from Fig 5(a) that the susceptible normal cells re-grow to the carrying capacity when the amount of viral particles reduces. From Fig 4, it can be observed that administering the viral doses at successive time points leads to rapid reduction of both susceptible normal and tumor cell populations.

### Tolerable normal cell depletion

In conjunction with the **Brief guidelines for *R*_0_ analysis** in S1 Text, we investigate how much should normal cells be infected by the oncolytic virus in order to maximize tumor reduction. One plausible approach in which normal cells can augment oncolytic virotherapy is to allow the virus to infect some normal cells in the tumor site, given that the basic reproductive number of the virus is less than unity (i.e., *R*_0_ < 1).

In cancer treatment, white blood cell (WBC) count (which incorporates all circulating lymphocytes) is an important factor which is used to determine health status of a patient prior to treatment. Most importantly, in clinics, WBC count is a first diagnostic measure used to screen for potential virus infection [71]. In humans, the normal WBC count is in the range of approximately 5 × 10^9^−10^10^ cells/*μl* [71]. In our model, it is crucial to track a population of normal (healthy) cells because it is important not to deplete normal cells beyond tolerable losses. Thus, we need to determine a threshold, denoted by $\tilde{N_{S}}$ cells, at which normal cells should not be depleted. However, since it often difficult to delineate what population of normal (non-cancerous) cells constitute in clinics, in our model simulations, we link the population of normal cells with white blood cells. White blood cells are always present whenever there is infection. Since oncolytic viruses that are not 100% tumor specific can also infect non-cancerous cells (even white blood cells such as neutrophils and monocytes), we use WBC count as a measure of normal cell depletion resulting from oncolytic virus infection in the vicinity of tumor cells. More importantly, we track the normal cell population in order to determine a stage at which our hypothetical patient would no longer attain full remission from therapy. We assume the following relationship between normal cell population and WBC count:

$$\tilde{N_{S}}=\alpha_{1}B\;\;\text{and}\;\text{that}$$ (12)

$$\tilde{B}=fB_{0},$$ (13)

where $\tilde{N_{S}}$ denotes the total minimum number of normal (healthy) cells that should not be depleted in virotherapy, *α*_1_ denotes a constant fraction, $\tilde{B}$ denotes the cutoff level of white blood cell count for humans, below which treatment should cease, *f* denotes a constant fraction, and *B*_0_ denotes the initial normal WBC count prior to treatment. Here, we chose $\tilde{B}=10^{8}$ cells/*μl* and *B*_0_ = 4.2 × 10^10^ cells/*μl* are taken in [72]. Thus, we estimate $f=\tilde{B}/B_{0}=10^{8}/4.2\times 10^{10}=2.4\times 10^{-3}$. We estimate $\tilde{N_{S}}=fN_{S}^{0}=K_{N}\times \left(2.4\times 10^{-3}\right)=2.4\times 10^{9}$ cells, where $N_{S}^{0}=K_{N}$ denotes the carrying capacity of normal cells at the start of oncolytic virotherapy. Here, $\tilde{N_{S}}$ serves as the level at which our hypothetical patient would no longer attain full remission if the oncolytic therapy continues.

**Fig 6:**
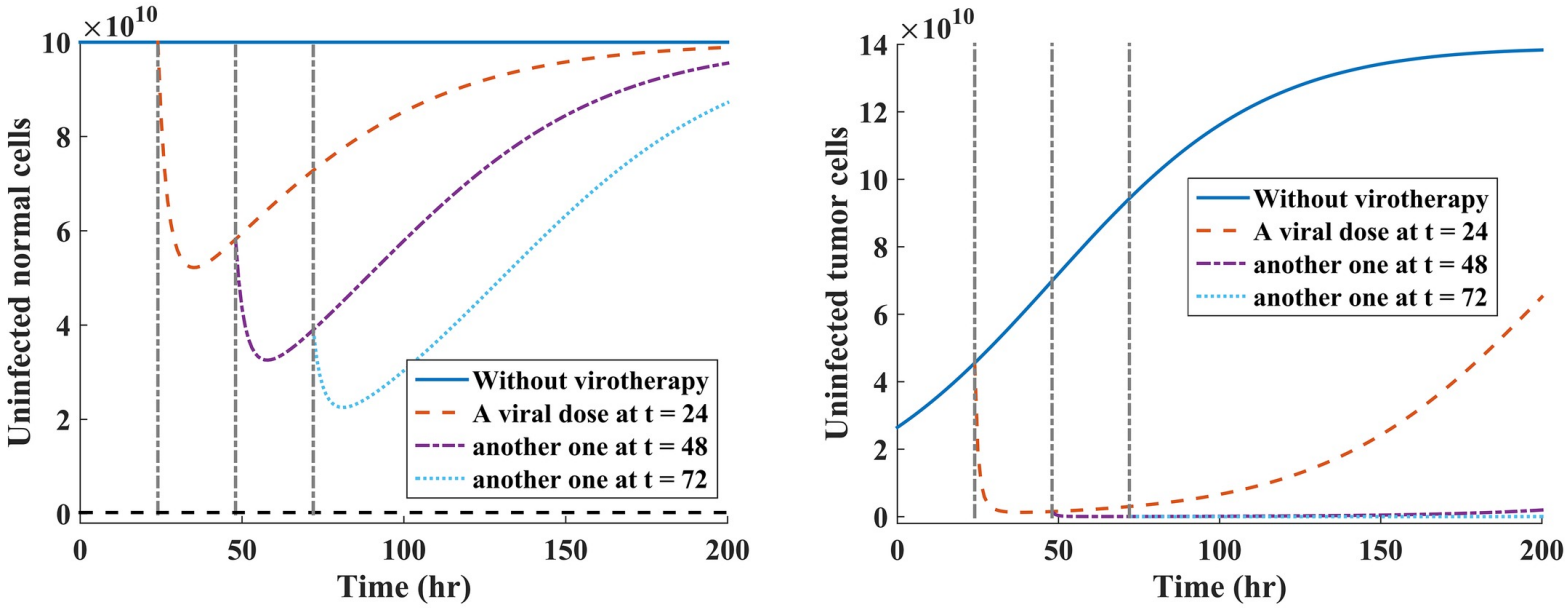
Scenario 1: Comparison of cell depletion under multi-viral dosing scheme. Fig 6(a) indicates reduction of normal cell population when $R_{0N}=\frac{\left(1-R_{0T}\right)}{4}$. Fig 6(b) shows reduction of tumor cells when $R_{0N}=\frac{3(1-R_{0T})}{4}$. The corresponding cell depletion profile is provided in Table 4.

**Fig 7:**
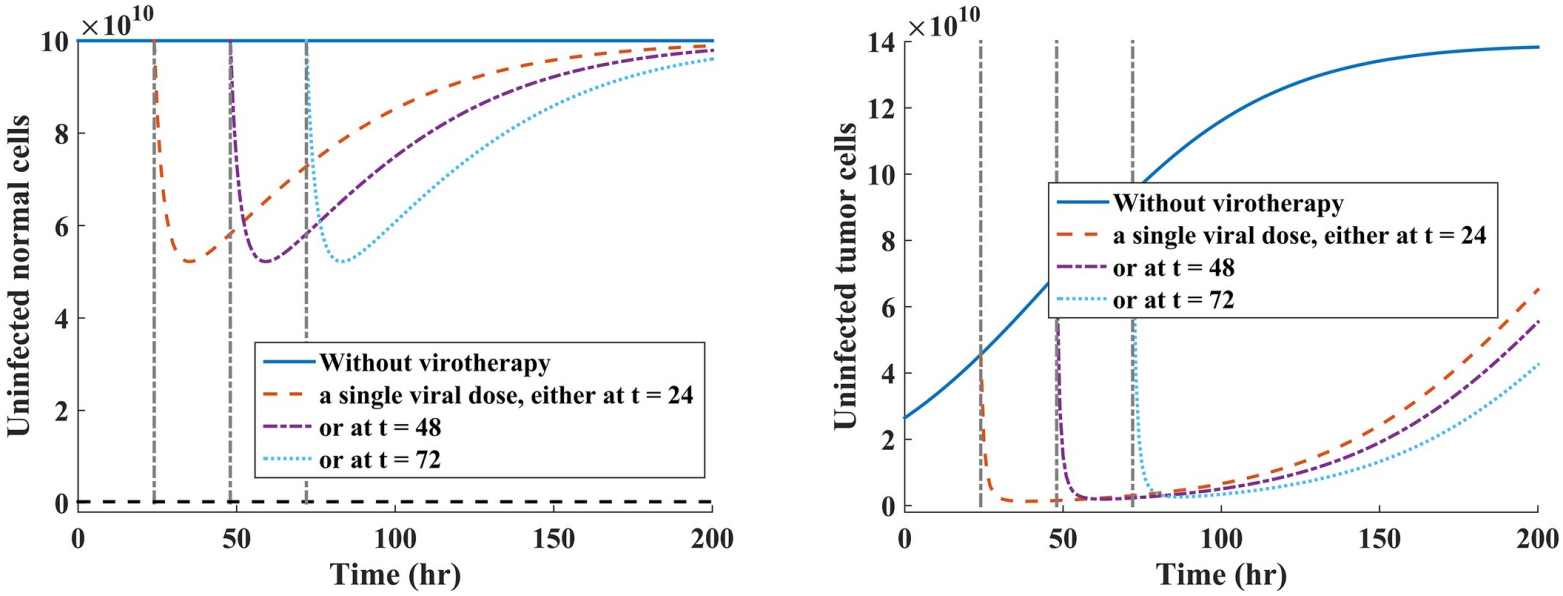
Scenario 2: Comparison of cell depletion under single-viral dosing scheme. Relative comparison of cell depletion when the oncolytic virus is administered at three distinct time points. Fig 7(a) indicates reduction of normal cell population when $R_{0N}=\frac{3(1-R_{0T})}{4}$. Fig 7(b) shows reduction of tumor cells when $R_{0N}=\frac{3(1-R_{0T})}{4}$.

We present results $\alpha =\frac{3}{4}$, shown in Figs 6 and 7. When $\alpha =\frac{1}{2}$, we obtained Fig 4 with corresponding cell depletion profile shown in Table 3. Note that when *α* = 0, then the oncolytic virus is 100% tumor-specific since *R*_0_ = *R*_0*N*_ + *R*_0*T*_.

**Table 3:** Minimum cell reduction achievable when *R*_0*N*_ = (1 − *R*_0*T*_)/2.

Time (hrs)	Normal cells	Tumor cells
Without therapy, *t* = 0	1 × 10^11^	2.647 × 10^10^
*t* = 24	7.2764 × 10^10^	9.0057 × 10^9^
*t* = 48	5.889 × 10^10^	2.2719 × 10^9^
*t* = 72	5.1158 × 10^10^	5.8419 × 10^8^
*t* = 96	4.6375 × 10^10^	1.5049 × 10^8^
*t* = 120	4.3228 × 10^10^	3.8686 × 10^7^
*t* = 144	4.1055 × 10^10^	9.9173 × 10^6^
*t* = 168	3.9494 × 10^10^	2.5356 × 10^6^
*t* = 192	3.8358 × 10^10^	6.4674 × 10^5^

**Fig 8:**
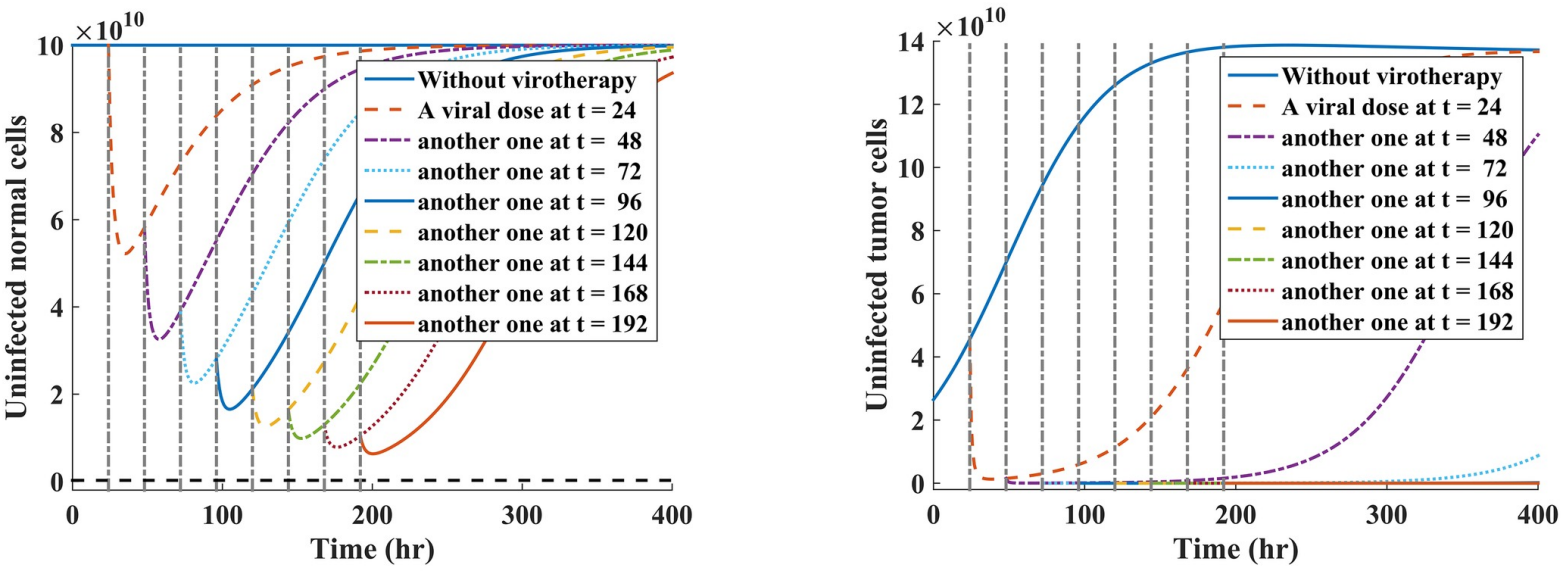
Simulation of cell depletion when $R_{0N}=\frac{3(1-R_{0T})}{4}$ under scenario 1. Fig 8(a) indicates a decline in normal cell population. Fig 8(b) shows the tumor shrinks down to zero over time.

As we would expect, under periodic dosing scheme, whenever *R*_0*N*_ = 3(1 − *R*_0*T*_)/4, it is possible to drive tumor population to extinction, shown in Fig 8(b) and Table 4, while minimizing much loss of normal cell depletion, shown in Fig 8(a) and Table 4.

**Table 4:** Minimum cell reduction achievable when *R*_0*N*_ = 3(1 − *R*_0*T*_)/4.

Time (hrs)	Normal cells	Tumor cells
Without therapy, *t* = 0	1 × 10^11^	2.647 × 10^10^
*t* = 24	5.22 × 10^10^	1.2889 × 10^9^
*t* = 48	3.2569 × 10^10^	4.3147 × 10^7^
*t* = 72	2.2536 × 10^10^	1.4405 × 10^6^
*t* = 96	1.6541 × 10^10^	4.7884 × 10^4^
*t* = 120	1.2607 × 10^10^	1.5857 × 10^3^
*t* = 144	9.8565 × 10^9^	5.2378 × 10^1^
*t* = 168	7.8477 × 10^9^	1.7291
*t* = 192	6.331 × 10^9^	0.057081

For all tested values of *R*_0_ < 1, we note that as long as $R_{0N}=\frac{3(1-R_{0T})}{4}$, the tumor was eliminated. Also, we note that increasing values of *R*_0*N*_ slightly, the tumor can still be controlled. Most importantly, we observe that multi-viral (periodic dosing) dosing schemes offers better results in terms of tumor cell depletion, shown in Fig 8. Thus, we compared minimum cell depletion for each cell population for varying values of *α* under scenario 1. Results are provided in Tables 3 and 4.

Notably, from Table 4, when *R*_0*N*_ is close to *R*_0*T*_, tumor cell population is diametrically reduced and become eliminated between time *t* = 168 and *t* = 192 hours. We note that at time *t* = 192 hours, there are 0.057081 cells because our model is based on delay differential equations (DDEs). Numerical solutions from the DDEs can only provide some information on the average behavior of the variables of the model; thus, complete tumor elimination cannot be guaranteed in our model. In principle, however, when the average number of cells is less than **one**, then we can assume that such cells are ideally eradicated. Hence the tumor cells are eradicated in this scenario. On the other hand, interestingly, we note that the population of normal cells has not reached the threshold value, $\tilde{N_{S}}=K_{N}\times \left(2.4\times 10^{-3}\right)=2.4\times 10^{9}$ cells, beyond which we expect our hypothetical patient not to attain complete tumor remission. Most importantly, our results indicate no toxicity to normal cells, since the minimum depletion of 6.331 × 10^9^ normal cells, at time *t* = 192 hours, is above the threshold value, $\tilde{N_{S}}$ cells. What these results suggest is that designing an oncolytic virus that is capable of exploiting a significant number of normal cells in the neighbourhood of the tumor, can plausibly drive tumor cells to extinction. Our results are more applicable the treatment scenario where tumors that cannot be reached directly.

## Discussion

In this work, we set out to answer the question of “How can oncolytic virus infection of some normal cells in the vicinity of tumor cells enhance oncolytic virotherapy?” To this end, we developed a delay differential equation model that describes the dynamics of the oncolytic virus that is not 100% tumor-specific on normal and tumor cell populations. A major focus of our model analysis was to explore and delineate the effects of oncolytic potency and specificity of viruses that not 100% tumor-specific in virotherapy. We now outline all the notable features of our model analyses and simulations that provide a comprehensive picture of the model evolution and behavior on how oncolytic viruses differentially exploit the populations of normal and tumor cells during oncolytic virotherapy.

**The oncolytic viral tumor-specificity.** From a mathematical point of view, we sought for the solutions of the model that provide a succinct framework on the oncolytic viral tumor-specificity that maximises tumor reduction while minimizing the undesirable toxicity on normal cell population surrounding the tumor. Most importantly, the model predicts the evolution of three non-trivial virus free steady states; the tumor-free steady state in which only normal cells are ultimately present, the tumor-only steady state in which only tumor cells are present, and the co-existence steady state in which both normal and tumor cells are present. The model equilibria analysis and simulations show that the coexistence steady state plays a crucial role in controlling viral infections on normal cell population at the onset of virotherapy. In particular, they show that whenever the basic reproductive number *R*_0_ < 1, infection of normal cells by the oncolytic virus may be tolerable only if such infections can aid to eliminate tumor cells (see Fig 8(b) and Table 4) that would otherwise be difficult.

We then examined differing trajectories of oncolytic virus infection on tumor cells and a limited number of normal cells. From the model simulations, Figs 5(a) and 4(a), we note that normal cells quickly self-regenerate after initial reduction. The attenuated damage on normal cells has distinct treatment explanations: (a) Direct viral oncolysis is limited by early induction of antiviral immune response, (b) Virus propagation is inhibited by beta interferon (IFN-*β*) that is often secreted by normal cells. On the other hand, we assume that infecting a limited portion of normal cell in the tumor bed with oncolytic virus could augment oncolytic virotherapy. Given that the virus can infect and replicate in normal cells, a progeny of infectious virions produced from lysed cells can further spread and infect other uninfected tumor cells. Whenever the basic reproductive number of normal cells is less than one, *R*_*ON*_ < 1, viral infections on normal cells would eventually stop, coupled by the fact that normal cells often rapidly inhibit virus propagation [47]. Our findings suggest that oncolytic viral infection of normal cells can be useful only when the virus replicates rapidly within infected normal cells.

Most interestingly, our results, from Table 4, indicates that oncolytic viruses that are capable of exploiting some normal cells, as their replication factories, can drive tumor cells to extinction within biologically reasonable time frame. We emphasize here that such oncolytic viruses should have a higher replication preferential profile, as illustrated by respective basic reproductive numbers in our model, to tumor cells than normal cells. It can be seen from Tables 4 and 3, that when the oncolytic virus exploits more normal cells within a given threshold, then tumor cell population is driven to extinction rapidly, as shown explicitly in Table 4. From clinical point of view, our theoretical results suggest that in normal cell population that can quickly self-renew (e.g., white blood cells or the liver), oncolytic virus infection on limited portion of normal cells may aid to eradicate tumor cells that would otherwise be difficult to eliminate. This is achievable and tolerable only if such viral infections are not endemic (i.e., the basic reproductive number of the virus is less than unity, *R*_0_ < 1).

**The effects of the potential antitumoral and antiviral immune responses in oncolytic virotherapy.** Global sensitivity analysis elucidates that the model is very sensitive to a number of parameters at the initial dose (i.e., at time *t* = 24 hours). For tumor cell population, proliferation rate of uninfected tumor cells, *r*_*T*_, is the most positively correlated parameter with the viral particles. At this early stage of tumor development, as the proliferation rate of susceptible tumor cells, *r*_*T*_, increases, tumor density will also increase. This observation is conformable with other findings that tumor cell proliferation is a major essential factor for benign tumors, particularly the malignant tumors [73]. Note that the susceptible tumor cell population would only decrease if virus replication outpaced the intrinsic tumor growth rate. This observation is in agreement with the simulation results in Figs 4(b) and 5(b) which indicates rapid reduction of the susceptible tumor cell population when the VSV doses are administered periodically (i.e., at time *t* = 24). Note that for all time points of viral dose (see Fig 3), lysis rate of susceptible tumor cells by tumor-specific immune cells (*γ*_*T*_) is the major determinant parameter in the model. Interestingly, this result confirms the idea that a success of an oncolytic virotherapy does not only depend on direct oncolysis but also on the influence of immune response against tumor cells [45].

For normal cells, we similarly interpret the positive and negative correlations between the parameters of normal cells and the oncolytic viral particles. The sensitivity analysis reveals that the model is highly sensitive to the lysis rate of the infected normal cells by virus-specific immune cells (*γ*_*V*_) in first viral dose time point (i.e., at time *t* = 24 hours). This observation suggests that initial viral infection of normal cells, can quickly induce antiviral immune response against the infected cells.

**Assessing the effectiveness of treatment strategies on tumor and normal cells after injection with oncolytic virus.** We investigated the effects of two treatment strategies in oncolytic virotherapy: one single-viral dose illustrated in Fig 5, where viral dose is administered at three independent times, and multiple-viral doses (i.e., periodic dosing schedule) shown in Fig 4, where the virus is given at three successive times. The value of *R*_0_ provides useful insights on the dynamics of oncolytic viral infection on normal and tumor cell populations because: (a) Whenever the basic reproductive number of the model is less than one (*R*_0_ < 1), the oncolytic viral replication occurs in both normal and tumor cell populations; (b) When *R*_0_ < 1, viral infections on normal cells might trigger antiviral immune response against the infected cells [66]. From Fig 5, we note that single-viral dosing strategy reduces susceptible normal cells by same amount, irrespective of the time of dosing, and the cells are rapidly self-renewing. Similarly, this strategy yields similar results with respect to tumor cells. The cell count of the susceptible cell population is quickly reduce, and followed by a rapid tumor relapse. In Fig 4, we note that multiple-viral dosing (i.e., periodic dosing) has a significant effect on the susceptible tumor cell population than on normal cell population. This strategy suggests that continued periodic dosing may eradicate the tumor or at least delay tumor growth. Comparing these two therapeutic strategies, we note that multiple-viral dose regime, shown Fig 4, offers more favorable treatment outcomes than the single-viral dose regime, shown Fig 5, with respect to reduction of susceptible tumor cell population.

Our model results are comparable with other mathematical models. Our model predicts that the oncolytic virus (such as VSV) that lyses infected cells fast, may drive tumor cell population to extinction rapidly. This finding is consistent with the model by Wein et al. [74] who modeled tumor-virus dynamics using a system of partial differential equations. Indeed, evidence indicates that if the oncolytic virus kills infected cells fast, then the progeny of new virions has a chance to spread and infect other uninfected tumor cells, prior to accumulation of adaptive immunity [67]. Otherwise, the induced adaptive immune response would then eradicate both infected and the remnant uninfected tumor cells. Furthermore, our computational results also conform with results from Okamoto et al. [23] in that oncolytic virus infection of some normal cells can facilitate tumor control. Even better, our computational results, illustrated in Table 4 and Fig 8(b), indicate that tumor cell population can quickly be eradicated whenever the oncolytic virus exploits a significant amount of normal cells above a given acceptable threshold value. It is also shown that tumor burden can at least be reduced, as indicated in Table 3, but not completely eradicated within the given biological time frame.

Our results suggest that when designing an oncolytic virus that is not 100% tumor-specific, it is important to consider viral dosing scheduling (with respect to time and frequency of dosing) because oncolytic viral infections on normal cells might yield desirable or undesirable outcomes in virotherapy. This could be seen from Fig 5, for single dosing schedule, and Fig 4, for multiple dosing schedule, that different dosing strategies provide different outcomes. From the sensitivity analysis, our results suggest that when developing the oncolytic virus that is not 100% tumor-specific, it is important to note that viral infections on normal cells could lead to early induction of antiviral immune response that might inhibit further viral propagation.

## Conclusion

In conclusion, our mathematical model shows that viral infections on normal cells can indeed augment oncolytic virotherapy if the virus replicates fast within the infected cells. Our results may be useful in the discovery of new oncolytic viruses or attenuation of known wild viral strains, such wild-type oncolytic VSV [32] or VSV variants. Results of our global sensitivity analysis have provided invaluable insights about the parameters that influence growth kinetics and tumors’ response to oncolytic virus, and the adaptive immune response. Our findings support the design of oncolytic viruses that is not 100% tumor-specific, but have higher oncolytic potency towards tumor cells than normal cells, and have high capacity to recruitment adaptive antiviral and antitumoral immune responses. We believe our work opens new possibilities for designing new attenuated oncolytic viruses that can be examined in a clinical setting under complex scenarios in which tumors cannot be reached directly.

Finally, an important model extension would be to account for spatial intratumoral (within tumor) heterogeneity. It is known that that tumor heterogeneity can affect virus diffusivity within tumor cells. Another interesting possible of the model extension would be to account for variations of the immune responses towards infected and uninfected tumor cells. Currently, our model does not account for varying tumor immune responses when implementing experimental oncolytic viral dosages. Pre-existing antiviral immune responses, when treating patients who were exposed to oncolytic virotherapy before, may result in differing treatment response rates in the clinic.

## Supporting information

S1 Text Supplemental information. Contents: 1) Parameter estimation. 2) Model Basic Reproductive Number. 3) Stability analysis of the virus free steady states. 4) MATLAB Syntax for the ODE system counterpart of the model.
(PDF)
