# Supplementary material for: Oncolytic potency and reduced virus tumor-specificity in oncolytic virotherapy. A mathematical modelling approach
Source: PLoS One. 2017 Sep 21;12(9):e0184347. doi: 10.1371/journal.pone.0184347 (PMC5608221; doi:10.1371/journal.pone.0184347)
Supplement: S1 Text — Contents: 1) Parameter estimation. 2) Model Basic Reproductive Number. 3) Stability analysis of the virus free steady states. 4) MATLAB Syntax for the ODE system counterpart of the model. (PDF) [file pone.0184347.s001.pdf]

Supplemental Information

Oncolytic Potency and Reduced Virus Tumor-specificity in Oncolytic Virotherapy. A Mathematical Modeling Approach

Khaphetsi Joseph Mahasa[1*], Amina Eladdadi[2], Lisette de Pillis[3], and Rachid Ouifki[4]
[1] DST/NRF Centre of Excellence in Epidemiological Modelling and Analysis (SACEMA), University of Stellenbosch, Stellenbosch, South Africa
[2] The College of Saint Rose, Albany, NY, USA
[3] Harvey Mudd College, Claremont, CA, USA
[4] Department of Mathematics and Applied Mathematics, University of Pretoria, Pretoria, South Africa

Contents

Parameter estimation 1

Model Basic Reproductive Number 2

Stability analysis of the virus free steady states 4

MATLAB Syntax for the ODE system counterpart of the model 7

Parameter estimation

To analyse and simulate our model, we determine the baseline parameter values from the literature that most correspond to available experimental data and biological facts. Since most of the available parameter values from the literature are reported in daily rates, we rescaled such parameter values by dividing each of them with $d * 24$ hours, where d denotes the number of days, to convert daily rates to hourly rates.

Susceptible normal cells. Uninfected normal cell proliferation rate, and the normal cell carrying capacity, $r_N = 0.00275$ hr^{-1} and $K_N = 10^{11}$ cells, has been respectively taken from [1]. Since wild-type vesicular stomatitis virus can infect normal cells, the rate at which it infects normal cells, β_N, is not known precisely. However, for our modeling purpose, the hourly infection rate $\beta_N = (1.7 \times 10^{-8})/24$ virion^{-1} hr^{-1} of normal cells is rescaled from Friedman et al. [2].

Susceptible tumor cells. Similarly, we have taken the proliferation rate, $r_T = 0.003$ hr^{-1}, and the tumor cell carrying capacity, $K_T = 1.47 \times 10^{11}$ cells, from [1]. The baseline value of the rate at which VSV infects tumor cells, $\beta_T = 0.038/24$ virion^{-1} hr^{-1}, has been rescaled from the daily rate in Eftimie et al. [3]. This parameter value is within the range $(5 \times 10^{-12.5}, 5 \times 10^{14})$

*Corresponding author email: mahasa@aims.ac.za

virion^{-1} hr^{-1} defined in [1], where the authors found out that the range allows for tumor persistence after the delay of 7 days prior to accumulation of the adaptive immune response. The lysis rate of susceptible tumor cells by tumor-specific immune cells, $\gamma_T = 1/24$ hr^{-1}, has also been rescaled from daily rate in Eftimie et al. [3]. The half-saturation constant of the tumor-specific immune cells that maintains half the maximum killing rate, $h_T = 40$ cells, has been taken from [3].

Infected normal cells. The death rate of infected normal cells, $\lambda_N = 1/24$ cells hr^{-1}, is an *ad hoc* value and has been chosen to conform with plausible biological outcomes. The rationale for this parameter value was based on the fact that the average time for an infected cell to undergo lysis is one day [2, 4, 5]. Similarly, the lysis rate of the infected normal cells by virus-specific immune cells, $\gamma_V = 1/24$ cells hr^{-1} is also an *ad hoc* value. This value is chosen based on the reasoning that the virus-specific immune cells do not distinguish between normal or tumor cells because they are recruited in response to viral antigens expressed by infected cells [6, 7].

Infected tumor cells. Similar to normal cells, the death rate of infected tumor cells due to VSV lysis, $\lambda_T = 1/24$ cells hr^{-1}, has been rescaled from daily rate in Eftimie et al. [3].

Oncolytic virus. The burst size of VSV from lysed infected tumor cells, $b_T = 1350$, is taken from [1]. For normal cells, we estimate that the oncolytic vesicular stomatitis virus (VSV) yields the burst size of $b_N = 1000$. This value was chosen based on the fact that VSV infection in normal cells is usually hampered by the presence of the interferon (IFN-β or $-\alpha$) [8]. Hence we chose $b_N \leq b_T$ since tumor cells are known to acquire deficiencies in antiviral inhibitory mechanisms [9, 10]. The clearance of the free virus particle by tumor-specific immune cells, $\omega = 2.5 \times 10^{-2}$ hr^{-1} was taken from [2, 11].

Tumor-specific immune cells. The hourly proliferation rate of tumor-specific immune cells in response to tumor antigens, $p_T = 0.0375/24$ hr^{-1}, was taken and rescaled from the daily rate in de Pillis et al. [12]. Assuming that the tumor-specific immune cells (i.e., tumor-specific CD8$^+$ T cells) have a half-life of 77 days as shown in [13], we estimate the hourly death rate of the tumor-specific immune cells, δ_T, to be $\delta_T = \frac{ln(2)}{(77\times24)} \approx 3.75 \times 10^{-4}$ hr^{-1}.

Virus-specific immune cells. We chose the *ad hoc* value of the proliferation rate of virus-specific immune cells in response to VSV antigens, $p_V = 0.025$ hr^{-1}, since it is the lower bound of the daily interval rate of the virus-specific immune cell proliferation rate shown by Eftimie et al. [3]. We tentatively chose this lower bound value because, during viral propagation within the infected cells, we assume that the immune response against the infected cells would be mainly driven by debris of infected cells since VSV has fast replication cycle [14]. Finally, the hourly death rate of the virus-specific immune cells, $\delta_V = 0.133/24 \approx 5.54 \times 10^{-3}$ hr^{-1} was rescaled from daily rate in Eftimie et al. [3].

Model Basic Reproductive Number

A basic reproductive number is defined as the average number of new infections generated by one infected cell, via cell lysis, during virotherapy in a completely susceptible cell population [15]. In general, if $R_0 > 1$, then, on average, the number of new infections resulting from one infected cell is greater than one. Thus, viral infections will persist in both normal and tumor cell populations. If $R_0 < 1$, then, on average, the number of new infections generated

by one infected cell in virotherapy is less than one. This implies that the viral infections will eventually disappear from the cell populations. Here, we provide a detailed description of the calculation of the basic reproductive number of the model. We use the next generation matrix approach [15, 16].

Proposition 1. *The basic reproductive number of model is given by*

$$R_0 = R_{0N} + R_{0T}$$

where

i. $R_{0N} := \frac{b_N \beta_N N_S}{\omega}$, *represents the basic reproductive number of the virus when introduced into a population of normal cells only*

ii. $R_{0T} := \frac{(Y_T + h_T) b_T \beta_T \lambda_T T_S}{((Y_T + h_T)\lambda_T + Y_T \gamma_T)\omega}$, *represents the basic reproductive number of the virus when introduced into a population of cancer cells only.*

Proof. By formally applying the next generation method, we determine the threshold parameter R_0 at a virus free equilibrium point $E_{NT} := (N_S, 0, T_S, 0, 0, 0, Y_T)$. After identifying the vectors of new infections and that of other transfers, we calculate the Jacobian matrices evaluated at the virus free equilibrium E_{NT}, and obtain

$$M = \begin{bmatrix} 0 & 0 & \beta_N N_S \\ 0 & 0 & \beta_T T_S \\ 0 & 0 & 0 \end{bmatrix}$$

$$N = \begin{bmatrix} \gamma_V Y_V + \lambda_N & 0 & 0 \\ 0 & \gamma_V Y_V + \lambda_T + \frac{\gamma_T Y_T}{h_Y + Y_T} & 0 \\ -b_N \lambda_N & -b_T \lambda_T & \omega \end{bmatrix}$$

The spectral radius of the matrix MN^{-1} is given by

$$R_0 = R_{0N} + R_{0T}$$

where

$$R_{0N} := \frac{b_N \beta_N N_S}{\omega}, \quad \text{and} \quad R_{0T} := \frac{(Y_T + h_T)\, b_T \beta_T \lambda_T T_S}{((Y_T + h_T)\,\lambda_T + Y_T \gamma_T)\,\omega}.$$

□

Brief guidelines for R_0 analysis. We aim to find a threshold in which the oncolytic viruses that can exploit both normal and tumor cells, such as vesicular stomatitis virus (VSV), can infect normal cells without much toxicity on normal cell population. The major goal of every oncolytic virus is to infect and lyse as many tumor cells as possible without much toxicity on the host normal tissue. The focus of our model analysis is centred around the basic reproductive number of the model. Numerical simulations, in conjunction with the analysis of the basic reproductive numbers, aim to shed light on design and use of oncolytic viruses that are not 100% tumor-specific. In particular, we seek for R_{0N} such that

$$R_{0N} + R_{0T} \simeq 1 \ \text{(but)} \ < 1.$$

Note that if, based on the value of R_{0N},

$$\begin{aligned} R_{0N} &\simeq 0 \ \text{ the virus cannot infect normal cells} \\ \text{or } &\gg 1 \ \text{ (the virus is too toxic on normal cells, hence not admirable.)} \end{aligned}$$

More importantly, R_{0N} should satisfy the following conditions:

$$\begin{aligned} R_{0N} &= \tilde{\alpha} R_{0T}, \quad \tilde{\alpha} \ll 1, \quad \text{where } \alpha \text{ is a small proportionality constant}, \\ R_{0N} &= \alpha(1 - R_{0T}), \quad \text{where } \alpha \text{ is a constant fraction.} \end{aligned}$$

And we also need that

$$\tilde{\alpha} = \frac{\alpha R_{0T}}{1 - R_{0T}}, \quad \text{and} \quad R_{0T} < 1.$$

With these guidelines on R_0, we investigate how the evolution of the oncolytic virus influences the treatment dynamics.

Stability analysis of the virus free steady states

Proposition 2. *The virus free equilibrium points E_N and E_T are always unstable, while E_{NT} is locally asymptotically stable if and only if $R_0 < 1$.*

Proof. The stability of E_{NT} is determined by the roots of following equation

$$(\delta_v + z)(zK_N - K_N r_N + 2N_S r_N)\left(P_2 z^2 + P_1 z + P_0\right)\left(Q_3 z^3 + Q_2 z^2 + Q_1 z + Q_0\right) = 0 \tag{1}$$

where

$$\begin{aligned} Q_3 :&= Y_T + h_T \\ Q_2 :&= (Y_T + h_T)\lambda_N + (Y_T + h_T)\omega + (Y_T + h_T)\lambda_T + Y_T\gamma_T \\ Q_1 :&= ((Y_T + h_T)\omega + (-Y_T - h_T) b_N\beta_N N_S + (Y_T + h_T)\lambda_T + Y_T\gamma_T)\lambda_N \\ &\quad + ((Y_T + h_T)\lambda_T + Y_T\gamma_T)\omega + (-Y_T - h_T) b_T\beta_T\lambda_T T_S \\ Q_0 :&= (((Y_T + h_T)\lambda_T + Y_T\gamma_T)\omega + ((-Y_T - h_T)\lambda_T - Y_T\gamma_T)) b_N\beta_N\lambda_N N_S \\ &\quad + (-Y_T - h_T) b_T\beta_T\lambda_N\lambda_T T_S \end{aligned}$$

and

$$\begin{aligned} P_2 &= \left(K_T{\delta_T}^2{T_S}^2 + 2K_T{\delta_T}^2 h_T T_S + K_T{\delta_T}^2{h_T}^2\right){h_Y}^2 \\ &\quad + \left(2K_T{T_S}^2\delta_T p_T + 2K_T T_S\delta_T h_T p_T\right)h_Y + K_T{T_S}^2{p_T}^2 \\ P_1 &= P_{13}T_S^3 + P_{12}T_S^2 + P_{11}T_S + P_{10} \\ P_0 &= P_{03}T_S^3 + P_{02}T_S^2 + P_{01}T_S + P_{00} \end{aligned}$$

with

$$
\begin{array}{rcl}
P_{13} & = & 2\xi^2 r_T = 2\xi a_0, \quad \xi = h_Y\delta_T + p_T \\
P_{12} & = & \xi\left[\left(-r_T + \delta_T + \gamma_T\right)K_T\xi + \left(4h_Tr_T - K_T\gamma_T\right)h_Y\delta_T\right] \\
 & = & \xi\left[b_0 + \delta_T\left(K_T\xi + 3h_Tr_Th_Y\right)\right] \\
P_{11} & = & \delta_Th_Th_Y\left[\left(2\delta_T + \gamma_T - 2r_T\right)K_T\xi + \left(2h_Tr_T - K_T\gamma_T\right)h_Y\delta_T\right] \\
 & = & \delta_Th_Th_Y\left[2b_0 + \left(2\delta_T - \gamma_T\right)K_T\xi + K_T\gamma_Th_Y\delta_T\right] \\
P_{10} & = & {\delta_T}^3{h_T}^2{h_Y}^2 + \delta_Th_Th_Yc_0
\end{array}
\tag{2}
$$

and

$$
\begin{aligned}
P_{03} &:= 2\delta_Tr_T\xi^2 \\
P_{02} &:= \delta_T\xi\left[\left(\gamma_T - r_T\right)\xi K_T + \left(4h_Tr_T - K_T\gamma_T\right)h_Y\delta_T\right] \\
P_{01} &:= 2{\delta_T}^2h_Th_Y\left[\left(\gamma_T - r_T\right)\xi K_T + \left(h_Tr_T - K_T\gamma_T\right)h_Y\delta_T\right] \\
P_{00} &:= -K_T{\delta_T}^3{h_T}^2{h_Y}^2r_T.
\end{aligned}
$$

Stability of E_T:
At the virus free and tumor endemic equilibrium point, E_T, we have $N_S = 0$ reducing the term $(zK_N - K_Nr_N + 2N_Sr_N)$ in the characteristic equation (1) to $(z - r_N)K_N$ which has $r_N > 0$ as a root. Hence E_T is unstable, implying that the tumor would persists growing uncontrollably.
Stability of E_N:
At the virus-and-tumor free equilibrium, E_N, we have $T_S = 0$ and $N_S = K_N$ reducing the term $\left(P_2z^2 + P_1z + P_0\right)$ in the characteristic equation (1) to $K_T\delta_T^2h_T^2h_Y^2\left(z + \delta_T\right)\left(z - r_T\right)$ which has a positive root r_T. Therefore, E_N is unstable. This condition means that normal cells are able to grow at an appreciable level in the absence of the tumor and virus. This result tend to highlight the significance of the ability of normal cells in continuing to maintain normal cell homeostasis in the absence of cancerous cells [17]. Note also that due to the choice of mass action infection kinetics in our model, viral replication does not affect the stability of this tumor free equilibrium.
Stability of E_{NT}:
At the virus free equilibrium with both tumor and normal cells, E_{NT}, we can see that P_2 is always positive. Let us show that P_1 and P_0 are positive.

$$
\begin{aligned}
P_1 = {} & 2\xi a_0T_S^3 + \xi\left[b_0 + \delta_T\left(K_T\xi + 3h_Tr_Th_Y\right)\right]T_S^2 \\
& + \delta_Th_Th_Y\left[2b_0 + \left(2\delta_T - \gamma_T\right)K_T\xi + K_T\gamma_Th_Y\delta_T\right]T_S \\
& + {\delta_T}^3{h_T}^2{h_Y}^2 + \delta_Th_Th_Yc_0.
\end{aligned}
$$

Using $a_0T_S^2 + b_0T_S = -c_0 = K_T\delta_Th_Th_Yr_T$, we obtain

$$
\begin{aligned}
P_1 = {} & \xi a_0T_S^3 + \xi K_T\delta_Th_Th_Yr_TT_S + \left(K_T\delta_T\xi^2 + 3h_Th_Y\delta_Ta_0\right)T_S^2 \\
& + \delta_Th_Th_Y\left[2b_0 + \left(2\delta_T - \gamma_T\right)K_T\xi + K_T\gamma_Th_Y\delta_T\right]T_S \\
& + {\delta_T}^3{h_T}^2{h_Y}^2 + \delta_Th_Th_Yc_0 \\
= {} & \xi a_0T_S^3 + \left(K_T\delta_T\xi^2 + h_Th_Y\delta_Ta_0\right)T_S^2 + 2h_Th_Y\delta_Ta_0T_S^2 \\
& + 2\delta_Th_Th_Yb_0T_S + \delta_Th_Th_YK_T\left[\left(2\delta_T - \gamma_T\right)\xi + \gamma_Th_Y\delta_T + \xi r_T\right]T_S \\
& + {\delta_T}^3{h_T}^2{h_Y}^2 - \delta_Th_Th_Yc_0.
\end{aligned}
$$

Furthermore, since $2h_T h_Y \delta_T a_0 T_S^2 + 2\delta_T h_T h_Y b_0 T_S = -2\delta_T h_T h_Y c_0$, then

$$\begin{aligned} P_1 &= \xi a_0 T_S^3 + \left(K_T \delta_T \xi^2 + h_T h_Y \delta_T a_0\right) T_S^2 - 3\delta_T h_T h_Y c_0 \\ &\quad + \delta_T h_T h_Y K_T \left(\left(2\delta_T + r_T - \gamma_T\right)\xi + \gamma_T h_Y \delta_T\right) T_S \\ &\quad + {\delta_T}^3 {h_T}^2 {h_Y}^2. \end{aligned}$$

Moreover, by using $b_0 = h_T h_Y r_T \delta_T + K_T \left(\xi\left(\gamma_T - r_T\right) - h_Y \gamma_T \delta_T\right)$, we obtain

$$\begin{aligned} P_1 &= \xi a_0 T_S^3 + \left(K_T \delta_T \xi^2 + h_T h_Y \delta_T a_0\right) T_S^2 - 3\delta_T h_T h_Y c_0 \\ &\quad + \delta_T h_T h_Y \left(2\delta_T \xi K_T + h_T h_Y r_T \delta_T - b_0\right) T_S \\ &\quad + {\delta_T}^3 {h_T}^2 {h_Y}^2 \\ &= \xi a_0 T_S^3 + \left(K_T \delta_T \xi^2 + 2h_T h_Y \delta_T a_0\right) T_S^2 - 2\delta_T h_T h_Y c_0 \\ &\quad - \delta_T h_T h_Y c_0 - h_T h_Y \delta_T a_0 T_S^2 \\ &\quad - \delta_T h_T h_Y b_0 T_S + \delta_T h_T h_Y \left(2\delta_T \xi K_T + h_T h_Y r_T \delta_T\right) T_S \\ &\quad + {\delta_T}^3 {h_T}^2 {h_Y}^2. \end{aligned}$$

Hence

$$\begin{aligned} P_1 &= \xi a_0 T_S^3 + \left(K_T \delta_T \xi^2 + 2h_T h_Y \delta_T a_0\right) T_S^2 - 2\delta_T h_T h_Y c_0 \\ &\quad + \delta_T h_T h_Y \left(2\delta_T \xi K_T + h_T h_Y r_T \delta_T\right) T_S + {\delta_T}^3 {h_T}^2 {h_Y}^2 > 0. \end{aligned}$$

We show next that $P_0 > 0$,

$$\begin{aligned} P_0 &= 2\delta_T \xi a_0 T_S^3 + \delta_T \xi \left[\left(\gamma_T - r_T\right)\xi K_T + \left(4h_T r_T - K_T \gamma_T\right) h_Y \delta_T\right] T_S^2 \\ &\quad + 2{\delta_T}^2 h_T h_Y \left[\left(\gamma_T - r_T\right)\xi K_T + \left(h_T r_T - K_T \gamma_T\right) h_Y \delta_T\right] T_S \\ &\quad - K_T {\delta_T}^3 {h_T}^2 {h_Y}^2 r_T. \end{aligned}$$

Since $\left(\gamma_T - r_T\right) K_T \xi + \left(h_T r_T - K_T \gamma_T\right) h_Y \delta_T = b_0$, then

$$\begin{aligned} &\xi \left[\left(\gamma_T - r_T\right)\xi K_T + \left(4h_T r_T - K_T \gamma_T\right) h_Y \delta_T\right] \\ &= \xi b_0 + 3\xi h_T r_T h_Y \delta_T \\ &= \xi b_0 + 3h_T h_Y \delta_T a_0, \end{aligned}$$

implying that

$$\begin{aligned} P_0 &= \delta_T \xi a_0 T_S^3 + \delta_T \xi T_S \left(a_0 T_S^3 + b_0 T_S\right) + 3h_T h_Y \delta_T^2 a_0 T_S^2 \\ &\quad + 2{\delta_T}^2 h_T h_Y b_0 T_S - K_T {\delta_T}^3 {h_T}^2 {h_Y}^2 r_T. \end{aligned}$$

Therefore,

$$\begin{aligned} P_0 &> 2h_T h_Y \delta_T^2 a_0 T_S^2 + 2{\delta_T}^2 h_T h_Y b_0 T_S - 2K_T {\delta_T}^3 {h_T}^2 {h_Y}^2 r_T \\ &= 2h_T h_Y \delta_T^2 \left(a_0 T_S^2 + b_0 T_S + c_0\right) = 0. \end{aligned}$$

Thus $P := P_2 z^2 + P_1 z + P_0 > 0$.
Concerning the polynomials Q_0, Q_1 and Q_2, we have Q_2 is always positive. Moreover,

$$
\begin{aligned}
Q_1 :&= ((Y_T + h_T)\,\omega\,(1 - R_{0N}) + (Y_T + h_T)\,\lambda_T + Y_T\gamma_T)\,\lambda_N \\
&\quad + ((Y_T + h_T)\,\lambda_T + Y_T\gamma_T)\,\omega\,(1 - R_{0T}) \\
Q_0 :&= (((Y_T + h_T)\,\lambda_T + Y_T\gamma_T)\,\omega\,(1 - (R_{0N} + R_{0T})))\,\lambda_N.
\end{aligned}
$$

Therefore,

i. If $R_0 := R_{0N} + R_{0T} > 1$, then $Q_0 < 0$ implying that the quadratic polynomial $Q := Q_2 z^2 + Q_1 z + Q_0$ has at least one root with positive real parts.

ii. If $R_0 < 1$, then $Q_0 > 0$. Moreover, we have $R_{0N} < 1$ and $R_{0T} < 1$ which implies that Q_1 is also positive. Since $Q_2 > 0$, then by the Routh-Hurwitz criterion [18, 19], the quadratic polynomial $Q := Q_2 z^2 + Q_1 z + Q_0$ does not have any roots with positive real parts. Hence, E_{NT} is locally asymptotically stable if and only if $R_0 < 1$, implying that the transient infections on normal and tumor cell populations would naturally be eliminated.

□

MATLAB Syntax for the ODE system counterpart of the model

```
function Virotherapy_Sing_Mult_Dose_ODE

    % This code is for the model dealing with generalist oncolytic viruses.
    % The model consists of a system of ODEs which are solved using the
    % solver ode23s.
    % The model investigates two main scenarios: 1. One single viral does
    % administred at various different times and 2. multiple viral dose
    % given at successive  time.
    % Event functions are also used to decide when to give the following
    % viral dose. These culd be based on: 1. The viral load reaching a certain
    % minimum (V(t) - 1e-6=0 for example), the rate of decline of tumor
    % cells is too slow (d(T_S+T_I)/dt + 1e-3 = 0 for example), or simply the
    % time since the last dose is too large (t-tDose(j)=1 week for
    % example).
    % The code creates folders for the plots whose name contain the current
    % dates, so we keep track of all the plots. It also creat a diray for
    % the model's results and discussion (if any), again, with dated names.

    global r_N K_N beta_N r_T K_T beta_T gamma_T h_T h_Y lambda_N gamma_V Params0
    global lambda_T b_T b_N omega p_V delta_V p_T delta_T tStart tEnd tSpan
    global aa bb cc R0N R0T R0  tDose V0 x01 x0

    beep off
    clc; close all
    set(0,'DefaultFigureWindowStyle','docked')
    set(0,'DefaultLineLineSmoothing','on');
```

```
set(groot,'DefaultFigureWindowStyle','docked',...
    'defaultLineLineWidth',2,...
    'defaultLineMarkerSize',4,...
    'defaultAxesFontSize',18,...
    'defaultAxesFontWeight','bold',...
    'defaultAxesFontName','Times New Roman');

mFileWorkingDirectory=pwd;
mFileParentDirectory=mFileWorkingDirectory;

% This is to creat subfolders for the plots and results. The folders'
% names include the current date and time to avoid overwritting them
format shortg
clk = datestr(datetime('now'),'dd-mmm-yy-HH');
PlotsFolder=['\ODE_PlotsDirectory-',clk];

% This is to avoid "creating" a folder that was exists already
if ~exist('ODE_PlotsDirectory', 'dir')
  mkdir(mFileParentDirectory, PlotsFolder);
end

% Same here: The results folder name containes the current date.
ResultsFolder=['\ODE_ResultsDirectory-',clk];
if ~exist('ODE_ResultsDirectory', 'dir')
  mkdir(mFileParentDirectory, ResultsFolder);
end

% The code's results go here. Anything that we would like to report
PlotsFullPath=[mFileParentDirectory,PlotsFolder];
ResultsFullPath=[mFileParentDirectory,ResultsFolder];
diary([ResultsFullPath,'\ODE_Results.txt']);
```

Model parameters

```
%     r_N = 0.00275;
%     K_N = 1e11;
%     r_T = 0.003;
%     K_T = 1.47e12;
%     beta_T = 5e-12;
%     beta_N = 1e-16;
%     gamma_T = 1/24;
%     gamma_V=1/24;
%     h_T = 40;
%     h_Y = 40;
%     lambda_N = 1/24;
%     lambda_T = 1/24;
%     b_T = 1350;
```

```
%      b_N=1e3;
%      omega = 2.5e-2;
%      p_V = 0.025;
%      delta_V = 5.54e-3;
%      p_T = 0.0375/24;
%      delta_T = 3.74e-4;
%     %%tau = 7;
```

Time span

```
tStart=0;
tEnd=400;
%tDose=[tStart,min(24,tEnd/4),min(48,tEnd/2),min(72,tEnd)];
tDose1=[tStart,24];
%tDose=[tStart,min(24,tEnd/8),min(48,tEnd/4),min(72,tEnd/2),min(96,tEnd)];
tDose=[tStart,min(24,tEnd/8),min(48,tEnd/4),min(72,tEnd/2),min(96,tEnd),...
min(120,tEnd),min(144,tEnd),min(168,tEnd),min(192,tEnd)];
tSpan = linspace(tStart,tEnd,1000);
```

Calculating the basic reproductive number: R_0

```
 aa=p_T*r_T+h_T*r_T*delta_T;
 bb=K_T*p_T*gamma_T+h_T*h_Y*r_T*delta_T-K_T*h_Y*r_T*delta_T-K_T*p_T*r_T;
 cc=-K_T*h_T*h_Y*r_T*delta_T;
 T_S=(-bb+sqrt(bb^2-4*aa*cc))/(2*aa);
 Y_T=p_T*T_S/(delta_T*(T_S+h_T));
 R0N=(b_N*beta_N*K_N)/omega;
 R0T=((b_T*beta_T*lambda_T*(h_T+Y_T)*T_S)/omega*((Y_T+h_T)*lambda_T + Y_T*gamma_T));
 R0 = R0N + R0T;
```

The model equations (ODE)

```
function dxdt = ModelEquations(~,x,Params)
    beta_N = Params(1);
    gamma_V = Params(2);
    b_N = Params(3);

    xSN=x(1);
    xST=x(2);
    xIN=x(3);
    xIT=x(4);
    xV=x(5);
    xYV=x(6);
    xYT=x(7);
    dxSNdt = r_N*xSN*(1-(xSN+xIN)/K_N) - beta_N*xSN*xV;
    dxSTdt = r_T*xST*(1-(xST+xIT)/K_T) - beta_T*xST*xV - gamma_T*...
             (xYT/(h_Y + xYT))*xST;
    dxINdt = beta_N*xSN*xV - lambda_N*xIN - gamma_V*xYV*xIN;
```

```
    dxITdt= beta_T*xST*xV - lambda_T*xIT - gamma_T*(xYT/(h_Y + xYT))*...
            xIT - gamma_V*xYV*xIT;
    dxVdt = b_T*lambda_T*xIT + b_N*lambda_N*xIN - omega*xV;
    dxYVdt = p_V*(xIT + xIN) - delta_V*xYV;
    dxYTdt = p_T*((xST + xIT)/(h_T + xST + xIT)) - delta_T*xYT;
    dxdt = [dxSNdt;dxSTdt;dxINdt;dxITdt;dxVdt;dxYVdt;dxYTdt];
end
```

Initial conditions

```
SN0 = K_N;
ST0 = 0.2*T_S;
NI0 = 0;
TI0 = 0;
YT0 = 0;
YV0 = 0;

x01 = [SN0;ST0;NI0;TI0;0;YV0;YT0];
x0 = NaN(7,length(tDose));
%X0 = NaN(7,length(tDose1)); % without viral dose
X0 = x01; % without viral dose
```

Model Solution

```
function Solutions = ModelSolution(t0,x0,Params)
    %options = ddeset('Events',@(t,x,y) myEvents(t,x,y,Params),'RelTol',1e-01,...
    %'AbsTol',1e-01);
    options0 = odeset('RelTol',1e-03,'AbsTol',1e-03);
    Solutions = ode23s(@(t,x) ModelEquations(t,x,Params),[t0 tEnd],x0,options0);
end
```

Model Solution without viral dose

```
function Solutions1 = ModelSolution1(t0,X0,Params)
    %options = ddeset('Events',@(t,x,y) myEvents(t,x,y,Params),'RelTol',1e-01,...
    %'AbsTol',1e-01);
    options0 = odeset('RelTol',1e-03,'AbsTol',1e-03);
    Solutions1 = ode23s(@(t,x) ModelEquations(t,x,Params),[t0 tEnd],X0,options0);
end
```

Evaluating the Model Solutions.

Parameters chosen for our simulations

```
Params0 = [beta_N,gamma_V,b_N];

% Viral injections used in our simulations. Changes these if the doses
% are not equal
```

```
V0=1e6*ones(size(tDose));
%V0=2e15*ones(size(tDose));


%Setting up empty arrays for the model solutions
Sol =NaN(1,length(tDose),7,length(tDose(1):tEnd));
SN = NaN(1,length(tDose),length(tDose(1):tEnd));
ST = NaN(1,length(tDose),length(tDose(1):tEnd));
IN = NaN(1,length(tDose),length(tDose(1):tEnd));
IT = NaN(1,length(tDose),length(tDose(1):tEnd));
V  = NaN(1,length(tDose),length(tDose(1):tEnd));
YV = NaN(1,length(tDose),length(tDose(1):tEnd));
YT = NaN(1,length(tDose),length(tDose(1):tEnd));
```

Model simulations.

Two scenarios are considered in these simulations: Scenario k=1 corresponds to a single viral dose administered at different times. while scenario k=2 represents successive viral injections administered according to the event function

```
for k=1:2
    for j=1:length(tDose)
        % Setting up the initial condition after each dose
        if j==1
            x0(:,j)=x01;
        else
            if k == 1
                % Here the initial condition for tDose(j) is equal to the
                % solution evaluated at tDose(j-1) Plus an additional viral
                % dose               %
                x0(:,j) = deval (ModelSolution(tDose(j-1),x0(:,j-1),Params0),...
                          tDose(j)) + [0;0;0;0;V0(j);0;0];
            else
                % Here the initial condition for tDose(j) is equal to the
                % initial condition x0 plus an additional viral dose
                x0(:,j) = deval (ModelSolution(tDose(1),x0(:,1),Params0),tDose(j))...
                         + [0;0;0;0;V0(j);0;0];
            end
        end

        %Evaluating the solutions for each dosing regim (tDoses) and each scenario
        Sol(k,j,:,tDose(j)+1:tEnd+1) = deval (ModelSolution(tDose(j),x0(:,j),...
                                       Params0),tDose(j):tEnd);
        SN(k,j,:) = Sol(k,j,1,:);
        ST(k,j,:) = Sol(k,j,2,:);
        IN(k,j,:) = Sol(k,j,3,:);
        IT(k,j,:) = Sol(k,j,4,:);
        V(k,j,:) = Sol(k,j,5,:);
```

```
        YV(k,j,:) = Sol(k,j,6,:);
        YT(k,j,:) = Sol(k,j,7,:);
    end
end
```

Events

```
function [value,isterminal,direction] = myEvents(t,x,y,Params)
    DerivSol=ModelEquations(t,x,y,Params);
    value = [DerivSol(2) + 1e-01,DerivSol(4) + 1e-01];
    isterminal = [0,0];
    direction = [-1,-1];
end
```

Plotting.
Changing directory for saving the plots

```
cd(PlotsFullPath);

% Line Styles
Styles={'-','--','-.',':','-','--','-.',':','-','--','-.',':'};

% Plots
for k=1:2

    % Figures' titles
    ttle=['R_{ON} = ', num2str(RON,'%10.0e\n'),', R_{OT} = ',...
          num2str(ROT,'%10.0e\n'),', Scenario = ', num2str(k)];

    % Figures' legends
    if k == 1
        MultiDose=repmat('another one at t = ',size(tDose(3:end),2),1);
        if length(tDose)==1
            lgd='Without virotherapy';
        elseif length(tDose)==2
            lgd=['Without virotherapy','A viral dose at t = ',num2str(tDose(2))]';
        else
            lgd=['Without virotherapy',['A viral dose at t = ...
                ',num2str(tDose(2))],cellstr([MultiDose,num2str(tDose(3:end)')])']';
        end
    elseif k == 2
        RepDose=repmat('or at t = ',size(tDose(3:end),2),1);
        if length(tDose)==1
            lgd='Without virotherapy';
        elseif length(tDose)==2
            lgd=['Without virotherapy','a single viral dose at t = ',...
                num2str(tDose(2))]';
        else
            lgd=['Without virotherapy',['a single viral dose, either at t = ...
```

```
                    ',num2str(tDose(2))],cellstr([RepDose,num2str(tDose(3:end)')])')]';
            end
        end
```

Returning to the working directory

```
cd(mFileWorkingDirectory);

end
```

References

[1] Okamoto KW, Amarasekare P, Petty IT. Modeling oncolytic virotherapy: Is complete tumor-tropism too much of a good thing? Journal of theoretical biology. 2014;358:166–178.

[2] Friedman A, Tian JP, Fulci G, Chiocca EA, Wang J. Glioma virotherapy: effects of innate immune suppression and increased viral replication capacity. Cancer research. 2006;66(4):2314–2319.

[3] Eftimie R, Dushoff J, Bridle BW, Bramson JL, Earn DJD. Multi-stability and multi-instability phenomena in a mathematical model of tumor-immune-virus interactions. Bulletin of mathematical biology. 2011;73(12):2932–2961.

[4] Kim PS, Crivelli JJ, Choi IK, Yun CO, Wares JR. Quantitative impact of immunomodulation versus oncolysis with cytokine-expressing virus therapeutics. Mathematical biosciences and engineering. 2015;12(4):841–858.

[5] Ganly I, Mautner V, Balmain A. Productive replication of human adenoviruses in mouse epidermal cells. Journal of virology. 2000;74(6):2895–2899.

[6] Bartlett DL, Liu Z, Sathaiah M, Ravindranathan R, Guo Z, He Y, et al. Oncolytic viruses as therapeutic cancer vaccines. Molecular cancer. 2013;12(1):1.

[7] Sobol PT, Boudreau JE, Stephenson K, Wan Y, Lichty BD, Mossman KL. Adaptive antiviral immunity is a determinant of the therapeutic success of oncolytic virotherapy. Molecular Therapy. 2011;19(2):335–344.

[8] Krishnamurthy S, Takimoto T, Scroggs RA, Portner A. Differentially regulated interferon response determines the outcome of Newcastle disease virus infection in normal and tumor cell lines. Journal of virology. 2006;80(11):5145–5155.

[9] Stojdl DF, Lichty B, Knowles S, Marius R, Atkins H, Sonenberg N, et al. Exploiting tumor-specific defects in the interferon pathway with a previously unknown oncolytic virus. Nature medicine. 2000;6(7):821–825.

[10] Keller BA, Bell JC. Oncolytic viruses–immunotherapeutics on the rise. Journal of Molecular Medicine. 2016;94(9):979–991.

[11] Paiva LR, Binny C, Ferreira SC, Martins ML. A multiscale mathematical model for oncolytic virotherapy. Cancer research. 2009;69(3):1205–1211.

[12] de Pillis LG, Radunskaya AE, Wiseman CL. A validated mathematical model of cell-mediated immune response to tumour growth. Cancer Research. 2005;65(17):7950–7958.

[13] de Pillis LG, Caldwell T, Sarapata E, Williams H. Mathematical Modeling of the Regulatory T Cell Effects on Renal Cell Carcinoma Treatment. Discrete and Continuous Dynamical Systems Series. 2013;18(4):915–943.

[14] Ayala-Breton C, Russell LOJ, Russell SJ, Peng KW. Faster replication and higher expression levels of viral glycoproteins give the vesicular stomatitis virus/measles virus hybrid VSV-FH a growth advantage over measles virus. Journal of virology. 2014;88(15):8332–8339.

[15] den Driessche PV, Watmough J. Reproduction numbers and sub-threshold endemic equilibria for compartmental models of disease transmission. Mathematical biosciences. 2002;180(1):29–48.

[16] Nowak M, May R. Virus dynamics: mathematical principles of immunology and virology. London: Oxford University Press; 2000.

[17] Ahmed AU, Rolle CE, Tyler MA, Han Y, Sengupta S, Wainwright DA, et al. Bone Marrow Mesenchymal Stem Cells Loaded With an Oncolytic Adenovirus Suppress the Anti-adenoviral Immune Response in the Cotton Rat Model. Molecular Therapy. 2010;18(10):1846–1856.

[18] Zi Z. Sensitivity analysis approaches applied to systems biology models. IET systems biology. 2011;5(6):336–346.

[19] Harris AL. Hypoxia–a key regulatory factor in tumour growth. Nature Reviews Cancer. 2002;2(1):38–47.
